# Binding to serine 65-phosphorylated ubiquitin primes Parkin for optimal PINK1-dependent phosphorylation and activation

**DOI:** 10.15252/embr.201540352

**Published:** 2015-06-26

**Authors:** Agne Kazlauskaite, R Julio Martínez-Torres, Scott Wilkie, Atul Kumar, Julien Peltier, Alba Gonzalez, Clare Johnson, Jinwei Zhang, Anthony G Hope, Mark Peggie, Matthias Trost, Daan MF van Aalten, Dario R Alessi, Alan R Prescott, Axel Knebel, Helen Walden, Miratul MK Muqit

**Affiliations:** 1 MRC Protein Phosphorylation and Ubiquitylation Unit, College of Life Sciences, University of DundeeDundee, UK; 2 Division of Biological Chemistry and Drug Discovery, College of Life Sciences, University of DundeeDundee, UK; 3 Division of Cell Signalling and Immunology, College of Life Sciences, University of DundeeDundee, UK; 4 College of Medicine, Dentistry & Nursing, University of DundeeDundee, UK

**Keywords:** Parkin, Parkinson’s disease, phosphorylation, PINK1, ubiquitin

## Abstract

Mutations in the mitochondrial protein kinase PINK1 are associated with autosomal recessive Parkinson disease (PD). We and other groups have reported that PINK1 activates Parkin E3 ligase activity both directly via phosphorylation of Parkin serine 65 (Ser^65^)—which lies within its ubiquitin-like domain (Ubl)—and indirectly through phosphorylation of ubiquitin at Ser^65^. How Ser^65^-phosphorylated ubiquitin (ubiquitin^Phospho-Ser65^) contributes to Parkin activation is currently unknown. Here, we demonstrate that ubiquitin^Phospho-Ser65^ binding to Parkin dramatically increases the rate and stoichiometry of Parkin phosphorylation at Ser^65^ by PINK1 *in vitro*. Analysis of the Parkin structure, corroborated by site-directed mutagenesis, shows that the conserved His302 and Lys151 residues play a critical role in binding of ubiquitin^Phospho-Ser65^, thereby promoting Parkin Ser^65^ phosphorylation and activation of its E3 ligase activity *in vitro*. Mutation of His302 markedly inhibits Parkin Ser^65^ phosphorylation at the mitochondria, which is associated with a marked reduction in its E3 ligase activity following mitochondrial depolarisation. We show that the binding of ubiquitin^Phospho-Ser65^ to Parkin disrupts the interaction between the Ubl domain and C-terminal region, thereby increasing the accessibility of Parkin Ser^65^. Finally, purified Parkin maximally phosphorylated at Ser^65^ *in vitro* cannot be further activated by the addition of ubiquitin^Phospho-Ser65^. Our results thus suggest that a major role of ubiquitin^Phospho-Ser65^ is to promote PINK1-mediated phosphorylation of Parkin at Ser^65^, leading to maximal activation of Parkin E3 ligase activity. His302 and Lys151 are likely to line a phospho-Ser^65^-binding pocket on the surface of Parkin that is critical for the ubiquitin^Phospho-Ser65^ interaction. This study provides new mechanistic insights into Parkin activation by ubiquitin^Phospho-Ser65^, which could aid in the development of Parkin activators that mimic the effect of ubiquitin^Phospho-Ser65^.

## Introduction

Mutations in genes encoding the protein kinase PTEN-induced kinase 1 (PINK1) and the ubiquitin E3 ligase, Parkin, are causal for early-onset Parkinson’s disease (PD) 1,2. Multiple lines of evidence indicate that these enzymes operate in a common mitochondrial signal transduction pathway 3,4. In mammalian cells, PINK1 is activated upon mitochondrial membrane potential depolarisation that can be induced by mitochondrial uncouplers, for example carbonyl cyanide m-chlorophenylhydrazone (CCCP), leading to mitochondrial recruitment and activation of Parkin 5-7,8-10. Biochemical and structural analysis has revealed that Parkin is autoinhibited and that conformational change would be required for its activation 11-13,14. Our own laboratory and that of others have revealed that PINK1 directly phosphorylates Parkin at serine 65 (Ser^65^) within its N-terminal ubiquitin-like (Ubl) domain 9,15 as well as the equivalent Ser^65^ residue of ubiquitin 16-18,19 and the phosphorylation of both of these residues is required for maximal activation of Parkin E3 ligase activity 16-18,19. However, the mechanism of how Ser^65^-phosphorylated ubiquitin (ubiquitin^Phospho-Ser65^) contributes to Parkin activation remains unknown.

Here, we report the discovery that ubiquitin^Phospho-Ser65^ can prime Parkin to be efficiently phosphorylated by PINK1 at Ser^65^ of its Ubl domain, which in turn leads to maximal activation of Parkin E3 ligase activity. We have investigated the interaction between Parkin and ubiquitin^Phospho-Ser65^ through biophysical and mutational analyses and identified that the conserved Parkin His302 and Lys151 residues play a critical role in ubiquitin^Phospho-Ser65^ binding to Parkin and for Ubl Ser^65^ phosphorylation by PINK1. Furthermore, we show that a His302Ala (H302A) mutation prevents optimal activation of Parkin by ubiquitin^Phospho-Ser65^ *in vitro*. In cell-based studies, we confirm that the H302A mutant leads to impaired Parkin Ser^65^ phosphorylation upon PINK1 activation induced by CCCP and moreover, immunofluorescence studies reveal that the Ser^65^-phosphorylated H302A mutant mainly resides in the cytosol in contrast to strong mitochondrial accumulation of Ser^65^-phosphorylated wild-type (WT) Parkin. We further show that maximally phosphorylated Ser^65^-WT Parkin exhibits robust E3 ligase activity that does not change following the addition of ubiquitin^Phospho-Ser65^. Overall, our data provide new insights into the activation of Parkin and suggest a two-step activation model wherein during Step 1, ubiquitin^Phospho-Ser65^ binds to Parkin inducing a conformational change of the Ubl domain that exposes Ser^65^ and in Step 2, PINK1 phosphorylates the Ubl domain more efficiently leading to maximal Parkin activation.

## Results and Discussion

### Ubiquitin^Phospho-Ser65^ primes Parkin Ser^65^ for phosphorylation by PINK1

**Figure 1:**
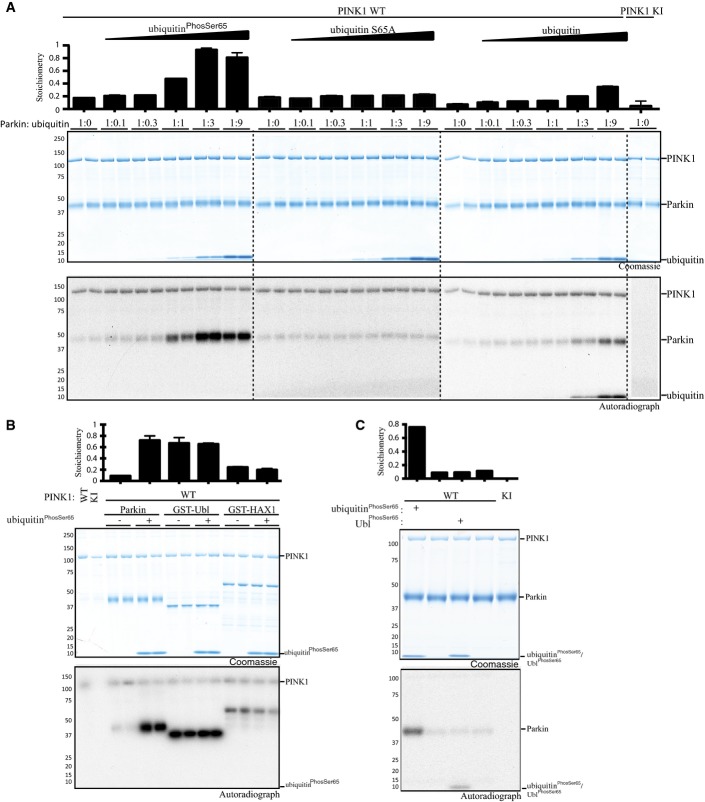
Ubiquitin^Phospho-Ser65^ primes Parkin for phosphorylation by PINK1 Ubiquitin^Phospho-Ser65^ enhances Parkin phosphorylation by PINK1. The effects of ubiquitin^Phospho-Ser65^ (left), wild-type (WT) (right) and Ser65Ala (S65A) (middle) ubiquitin on Parkin phosphorylation were investigated in a kinase assay. The indicated ubiquitin species was incubated with WT or kinase-inactive (KI) MBP-TcPINK1, Parkin and Mg^2+^ [γ-^32^P] ATP for 60 min. Parkin concentration was kept constant, whilst ubiquitin concentration was varied, reaching molar ratios indicated above the gel. Assays were terminated by the addition of LDS loading buffer, and products were analysed by SDS–PAGE. Proteins were detected by Colloidal Coomassie Blue staining (top panel), and incorporation of [γ-^32^P] ATP was detected by autoradiography (bottom panel). Data show mean of two trials ± s.d., *n* = 2 for each condition.

In previous work, we reported that PINK1 directly phosphorylates full-length Parkin at Ser^65^ within its Ubl domain. The rate of phosphorylation of full-length Parkin was substantially lower than that of the isolated Ubl domain, indicating that the Ser^65^ residue may be partially accessible in the full-length autoinhibited conformation 9. Strikingly, we observed that the addition of increasing amounts of ubiquitin^Phospho-Ser65^ markedly increased the rate as well as the stoichiometry of phosphorylation of the Parkin Ubl Ser^65^ residue reaching ∼0.9 in the presence of 3-fold excess of ubiquitin^Phospho-Ser65^ relative to Parkin, compared to ∼0.2 without (left panel, Fig1A). In contrast, we observed no enhancement of phosphorylation of Parkin by PINK1 in the presence of a non-phosphorylatable Ser65Ala mutant of ubiquitin (middle panel, Fig1A). Addition of increasing amounts of wild-type ubiquitin led to moderate enhancement of Parkin phosphorylation due to PINK1 phosphorylating ubiquitin and generating ubiquitin^Phospho-Ser65^ during the kinase reaction, which was confirmed by autoradiography (right panel, Fig1A). We did not observe any enhancement of phosphorylation of Parkin S65A as well as a Ubl-deleted fragment of Parkin (residues 80-465) by PINK1 in the presence of ubiquitin^Phospho-Ser65^ (Appendix Fig S1). This indicates that the ubiquitin^Phospho-Ser65^-mediated enhancement of Parkin phosphorylation by PINK1 is via Parkin Ubl Ser^65^ and not due to phosphorylation of additional sites on Parkin (Appendix Fig S1).

Selectivity of ubiquitin^Phospho-Ser65^-enhanced Parkin phosphorylation. Under similar conditions, the effects of ubiquitin^Phospho-Ser65^ on phosphorylation of various PINK1 substrates were assessed in a kinase assay, where equimolar amounts of Parkin, GST-Ubl and GST-HAX1 (all expressed in *E. coli*) were used in the presence or absence of ubiquitin^Phospho-Ser65^ and WT TcPINK1. The incorporation of radioactive [γ-^32^P] ATP into substrate proteins was measured and is displayed above the panels. Proteins were detected by Colloidal Coomassie Blue staining (top panel), and incorporation of [γ-^32^P] ATP was detected by autoradiography (bottom panel). Broken dividing lines indicate separate gels; asterisks denote the kinase band. The molecular mass in kDa is indicated. Data show mean of two trials ± s.d., *n* = 2 for each condition.

Ubiquitin^Phospho-Ser65^, but not Ubl^Phospho-Ser65^, enhances full-length Parkin phosphorylation by PINK1. Kinase assays in the presence or absence of ubiquitin^Phospho-Ser65^ or Ubl^Phospho-Ser65^ were carried out as in (A). Data are representative of two independent experiments.

We next investigated the specificity of ubiquitin^Phospho-Ser65^ enhancement of Parkin phosphorylation by PINK1. In parallel kinase assays, we compared the effect of ubiquitin^Phospho-Ser65^ on PINK1-dependent phosphorylation of Parkin, the isolated Ubl domain of Parkin and GST-HAX1 (Fig1B). HAX1 is a mitochondrial intermembrane protein, which we have recently identified as a good *in vitro* substrate of PINK1 although the physiological relevance has not yet been explored (Appendix Fig S2). We did not observe any effect of ubiquitin^Phospho-Ser65^ on PINK1’s ability to phosphorylate the isolated Ubl domain or HAX1, suggesting that ubiquitin^Phospho-Ser65^ does not affect PINK1 activity *per se* and that it has a specific effect on modulating the phosphorylation of full-length Parkin (Fig1B). We then tested whether isolated Parkin Ubl (1-76) phosphorylated at Ser65 (Ubl^Phospho-Ser65^) could also affect the stoichiometry of phosphorylation of full-length Parkin by PINK1 (Fig1C). In contrast to ubiquitin^Phospho-Ser65^, the addition of Ubl^Phospho-Ser65^ did not affect Parkin phosphorylation, indicating that this effect is phospho-ubiquitin specific (Fig1C).

Recent studies have demonstrated that PINK1 can phosphorylate poly-ubiquitin chains of different linkage types and lengths in addition to monomeric ubiquitin 19-21. We therefore investigated whether ubiquitin^Phospho-Ser65^-modified ubiquitin chains would have the same effect as monomeric ubiquitin in promoting Parkin phosphorylation by PINK1 (FigEV1A). Addition of ubiquitin dimers of each linkage type (Met1, Lys6, Lys11, Lys27, Lys29, Lys33, Lys48 and Lys63) or ubiquitin tetramers with Met1, Lys6, Lys11, Lys29, Lys33, Lys48 and Lys63 linkages 22,23 led to a similar enhancement of Parkin phosphorylation by PINK1 as that observed following the addition of monomeric ubiquitin (FigEV1A). To date, it is unknown whether PINK1 can efficiently phosphorylate monoubiquitin attached to a substrates. To address this, we employed a model monoubiquitylated substrate in which ubiquitin has been C-terminally fused to a Dac tag (a ∼28.5-kDa fragment of *E. coli* penicillin-binding Protein 5 comprising residues 37-297 that can be captured and released by binding to ampicillin-Sepharose) 24. We observed that Dac-ubiquitin could be readily phosphorylated by PINK1 leading to enhanced Parkin phosphorylation by PINK1 in a manner similar to monomeric ubiquitin (FigEV1B).

**Figure:**
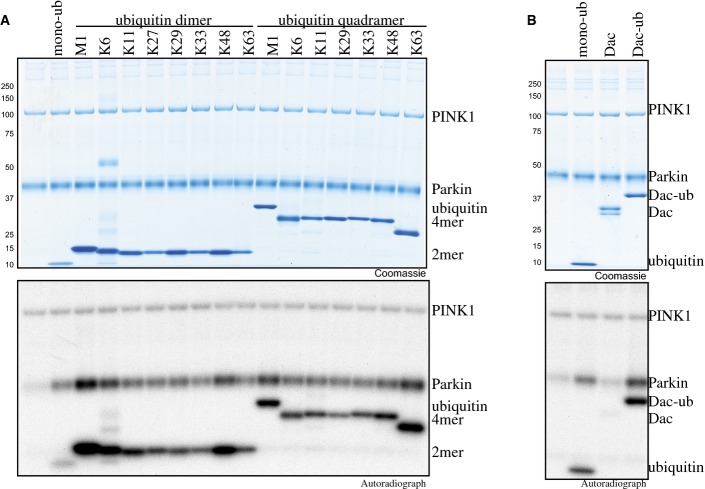
Ubiquitin dimers, tetramers or a mono-ubiquitylated substrate are capable of priming Parkin phosphorylation by PINK1 A, B (A) The effects of ubiquitin dimers of each linkage type (Met1 (M1), Lys6 (K6), Lys11 (K11), Lys27 (K27), Lys 29 (K29), Lys33 (K33), Lys48 (K48) and Lys63 (K63), ubiquitin tetramers with M1, K6, K11, K29, K33, K48 and K63 linkages and (B) the model mono-ubiquitylated substrate, Dac-ubiquitin (Dac-ub) were assessed in a PINK1 kinase assay similar to that in Fig1. Assays were analysed by SDS/PAGE. Proteins were detected by Colloidal Coomassie Blue staining (top panel), and incorporation of [γ-^32^P] ATP was detected by autoradiography (bottom panel).

In future work, it would be interesting to assess whether (multi)-monoubiquitylated substrates of Parkin, for example Miro1/2 or Mitofusin1/2 25,26, also become phosphorylated at ubiquitin Ser^65^ by PINK1 and whether these play a role in aiding in Parkin activation similar to what we have observed for Dac-ubiquitin. It would also be critical to define the interactions and timeline of PINK1 phosphorylation of free monomeric ubiquitin and poly-ubiquitin as well as mono/poly-ubiquitin attached to substrates that lead to an altered phospho-ubiquitome upon PINK1 and Parkin activation at the mitochondria.

### Identification of Parkin histidine 302 and lysine 151 as key residues required for binding and maximal activation of Parkin by ubiquitin^Phospho-Ser65^

**Figure 2:**
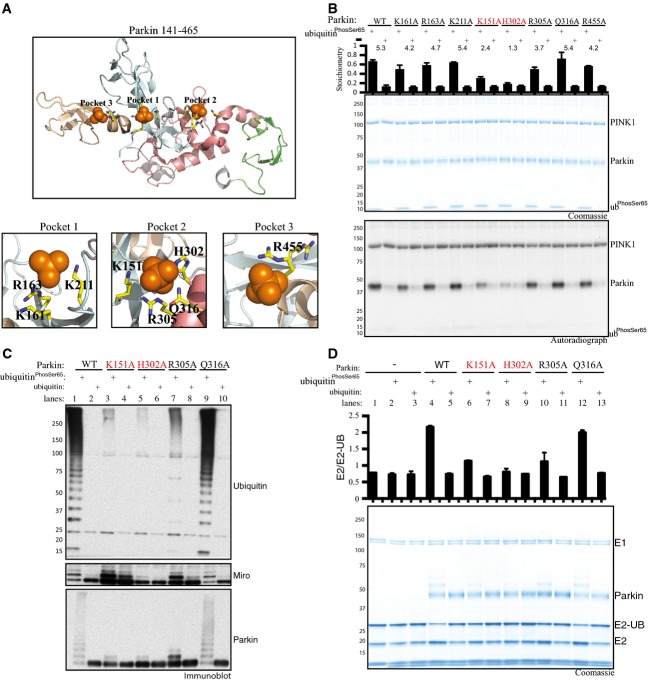
Identification of Parkin histidine 302 and lysine 151 as critical residues mediating ubiquitin^Phospho-Ser65^ interaction and activation of Parkin Structure of Parkin modified from 13 displaying the location of sulphate-containing pockets surrounded by the following residues: Pocket 1 (K161/R163/K211); Pocket 2 (K151/H302/R305/Q316); and Pocket 3 (R455).

We next investigated the potential interaction sites that enable ubiquitin^Phospho-Ser65^ binding and activation of Parkin E3 ligase activity. Previous structural analysis has highlighted that Parkin contains a putative phospho-Ser^65^-binding pocket that we term “Pocket 1” flanked by residues Lys161 (K161), Arg163 (R163) and Lys211 (K211) that lie within the RING0 domain 13 (Fig2A). Recent mutational analysis has suggested that these Pocket 1 residues are not required for binding of Parkin to ubiquitin^Phospho-Ser65^ 19. Upon inspection of the Parkin structure, we identified two further putative phospho-Ser^65^-binding pockets that we term “Pocket 2” and “Pocket 3” (Fig2A). Pocket 2 is formed from basic residues Lys151 (K151) (lies in RING0), His302 (H302), Arg305 (R305) and Gln316 (Q316) (that lie within a small loop between RING1 and IBR), whilst Pocket 3 is formed by a single residue Arg455 (R455) lying within the RING2 domain (Fig2A). To explore which residues were critical for ubiquitin^Phospho-Ser65^ function towards Parkin, we tested point mutants of all the putative Parkin pocket residues in a PINK1 kinase assay in the presence of ubiquitin^Phospho-Ser65^ (Fig2B). Strikingly, these data revealed that mutation of the His302 residue to Ala (H302A) in Pocket 2 almost completely prevented the ability of ubiquitin^Phospho-Ser65^ to enhance Parkin Ser^65^ phosphorylation by PINK1 (Fig2B). Furthermore, the K151A mutation in Pocket 2 also partially disrupted the ability of ubiquitin^Phospho-Ser65^ to modify the PINK1-dependent phosphorylation of Parkin, whereas the R305A mutant exhibited only a modest effect and Q316A no effect on ubiquitin^Phospho-Ser65^-mediated phosphorylation of Parkin Ser^65^ (Fig2B). Thermal shift analysis demonstrated that the Parkin H302A and K151A mutants are as stable as the wild-type Parkin with no measurable change in thermal stability, indicating no significant perturbation of fold or structural integrity (Fig EV2A). Since our assays are conducted with full-length Parkin and high-resolution structures of Parkin have been obtained using an N-terminal deleted fragment of Parkin (RING0-RBR domains), we cannot rule out the possibility that the topology of the ubiquitin^Phospho-Ser65^ pocket may be different in the full-length protein structure and involve additional amino acids not evident from the RING0-RBR structures. Nevertheless, our analysis strongly suggests a key role for H302 and K151 in ubiquitin^Phospho-Ser65^ action towards Parkin. Consistent with an important role, the H302 and K151 residues are highly conserved in multiple species of Parkin we have analysed (Appendix Fig S3).

His302 and Lys151 are critical for mediating ubiquitin^Phospho-Ser65^-enhanced phosphorylation of Parkin by TcPINK1. Wild-type (WT), full-length Parkin or the indicated Alanine mutant for each residue of the sulphate-containing pocket was incubated with wild-type (WT) MBP-TcPINK1, Parkin and Mg^2+^ [γ-^32^P] ATP in the presence or absence of ubiquitin^Phospho-Ser65^. Proteins were detected by Colloidal Coomassie Blue staining (top panel) and Parkin phosphorylation levels assessed by incorporation of [γ-^32^P] ATP detected by autoradiography (bottom panel) and displayed above the panel. Data show mean of two trials ± s.d., *n* = 2 for each condition.

Parkin His302Ala and Lys151Ala mutants disrupt ubiquitin^Phospho-Ser65^ mediated activation of Parkin E3 ligase activity. WT Parkin and putative Pocket 2 mutants (K151A, H302A, R305A, Q316A) were assessed for activity via ubiquitylation assays. Each reaction contained 0.05 mM ubiquitin comprising 25 μg of FLAG-ubiquitin (Boston Biochem) mixed with 5 μg of ubiquitin^Phospho-Ser65^ or non-phospho-ubiquitin. Parkin activity was evaluated by subjecting ubiquitylation reactions to analysis by SDS–PAGE and immunoblotting as follows: ubiquitin (anti-FLAG-HRP antibody), Parkin (anti-Parkin antibody) and Miro1 (anti-SUMO1 antibody). Data are representative of five independent experiments.

Parkin His302Ala and Lys151Ala mutants hinder Parkin’s ability to interact with ubiquitin^Phospho-Ser65^ and discharge ubiquitin from a loaded E2. WT Parkin and Pocket 2 mutants were assessed for their ability to discharge ubiquitin from a loaded UbcH7 (E2-Ub) enzyme with or without ubiquitin^Phospho-Ser65^ or non-phospho-ubiquitin. Reactions were subjected to SDS–PAGE analysis in the absence of any reducing agent. The activity was assessed by change in UbcH7 (E2)/UbcH7-Ub (E2-Ub) ratio. The quantification of Coomassie bands was performed by LICOR and is presented above the panel. Data show mean of three trials ± s.d., n = 1 for each condition.

**Figure:**
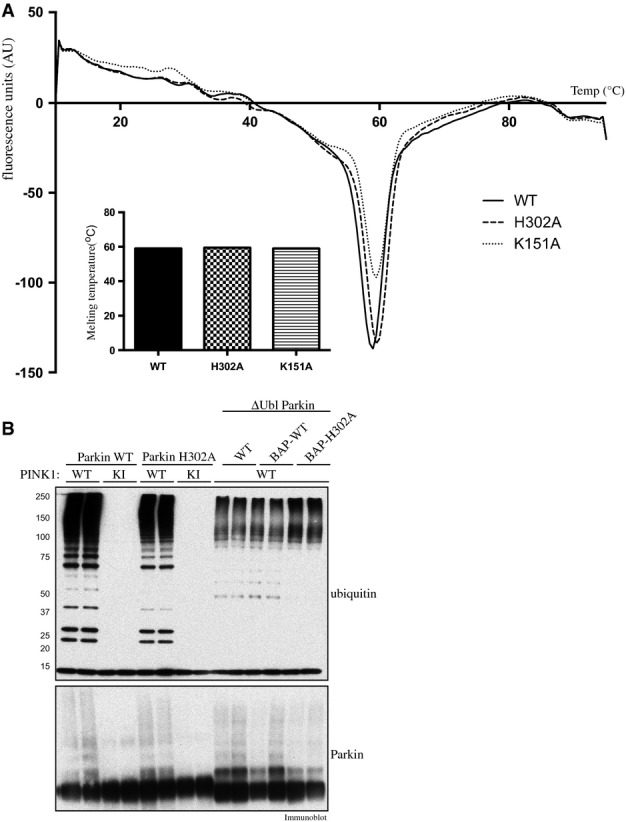
Analysis of stability and ubiquitin^Phospho-Ser65^-independent E3 ligase activity of “Pocket 2” mutants of Parkin Thermal denaturation curves obtained by differential scanning fluorimetry of wild-type (WT), and His302Ala (H302A)- and Lys151Ala (K151A)-mutant Parkin. Results are displayed as the differential of the fluorescence in arbitrary units divided by the differential of the temperature, plotted against temperature. Inset: the minimum of each curve indicates the melting point (Tm). Summary of melting points (Tm) of each protein as follows: WT (59°C); H302A (59.5°C); and K151A (59.5°C).

PINK1-dependent full-length Parkin E3 ligase activity mediated via phosphorylation of Ubl Ser65 and constitutive basal E3 ligase activity mediated by Ubl-deleted Parkin (ΔUbl; residues 80-465) are not affected by His302Ala (H302A) mutation. A 2 μg amount of wild-type full-length or ΔUbl-Parkin-biotin and H302A full-length or ΔUbl-Parkin was incubated with 1 μg of wild-type (WT), kinase-inactive (KI) or no TcPINK1 in an E3 ligase assay. Reactions were terminated after 60 min by the addition of LDS loading buffer and analysed by SDS/PAGE. Ubiquitin and Parkin were detected using anti-FLAG and anti-Parkin antibodies, respectively.

We next investigated the critical requirement of the H302 residue in ubiquitin^Phospho-Ser65^-mediated activation of Parkin E3 ligase activity. We have previously elaborated a robust Parkin E3 ligase activity that monitors the formation of free poly-ubiquitin chains, as well as the multi-monoubiquitylation of the substrate Miro1 26. Employing this assay, we observe marked activation of full-length Parkin E3 ligase activity when incubated with molar excess amounts of ubiquitin^Phospho-Ser65^ as judged by the formation of free poly-ubiquitin chains, Miro1 multi-monoubiquitylation and Parkin autoubiquitylation (lanes 1–2, Fig2C). Consistent with our phosphorylation assay analysis, we observed significant inhibition of the ability of ubiquitin^Phospho-Ser65^ to activate Parkin following mutation of His302, emphasised by a complete absence of Miro1 ubiquitylation and substantial reduction of free poly-ubiquitin chain formation and Parkin autoubiquitylation (lanes 5–6, Fig2C). In contrast, the H302A mutant did not disrupt the ability of TcPINK1 to induce Parkin E3 ligase activity via phosphorylation of Ubl Ser^65^ in the presence of a non-phosphorylatable Ser65Ala mutant of ubiquitin, suggesting that the H302A mutant is not significantly perturbing the conformation of Parkin and instead is selectively affecting ubiquitin^Phospho-Ser65^ interaction (Fig EV2B). Consistent with our previous phosphorylation analysis (Fig2B), the K151A mutant exhibited partial reduction in Parkin activity as judged by Parkin autoubiquitylation and free poly-ubiquitin chain formation (lanes 3–4, Fig2C) and the R305A mutant exhibited modest effects on Parkin activation upon addition of ubiquitin^Phospho-Ser65^ (lanes 7–8, Fig2C).

We next assessed the impact of the Pocket 2 mutants on Parkin activity using an E2 discharge assay that measures the ability of ubiquitin^Phospho-Ser65^ to stimulate the discharge of the ubiquitin-loaded E2, UbcH7, by Parkin. Whilst the addition of ubiquitin^Phospho-Ser65^ but not ubiquitin led to maximal E2 ubiquitin discharge by wild-type Parkin (lanes 4 and 5, Fig2D), this was completely disrupted by the H302A mutant (lanes 8 and 9, Fig2D) and partially by mutations of K151A (lanes 6 and 7, Fig2D) and R305A (lanes 10 and 11, Fig2D).

**Figure 3:**
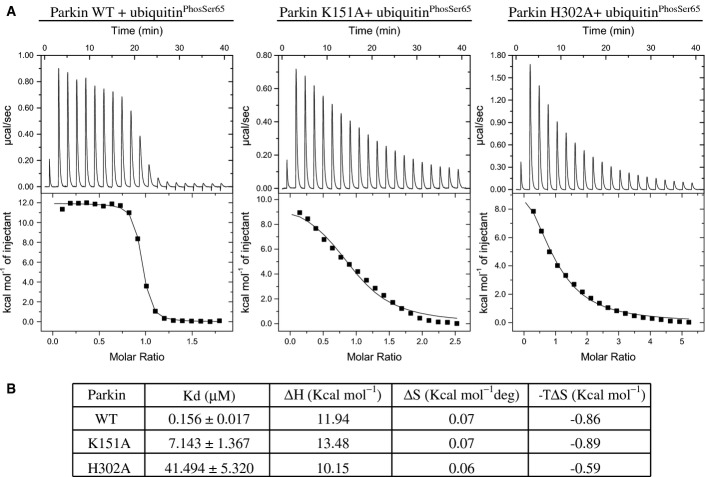
Parkin His302 and Lys151 are required for optimal binding with ubiquitin^Phospho-Ser65^ Parkin H302A and K151A mutants exhibit marked reduction in binding to ubiquitin^Phospho-Ser65^ compared to wild-type (WT) Parkin. Isothermal calorimetry analysis of Parkin WT (left), K151A mutant (middle) and H302A mutant (right) with ubiquitin^Phospho-Ser65^.

To demonstrate any direct involvement of Pocket 2 residues in binding of ubiquitin^Phospho-Ser65^, we undertook isothermal titration calorimetry (ITC) analysis of ubiquitin^Phospho-Ser65^ with full-length wild-type Parkin and the two most deleterious mutants H302A and K151A based on our previous analyses (Fig2B–D). Similar to a recent study, we found wild-type Parkin binds ubiquitin^Phospho-Ser65^ with a Kd of ∼156 nM affinity (left panel, Fig3A and B) 19. In contrast, we observed the K151A mutant binds ubiquitin^Phospho-Ser65^ with 45-fold lower affinity (K_d_ of ∼7 μM) than wild-type Parkin (middle panel, Fig3A and B) and the H302A mutant exerted a more dramatic effect with a 265-fold reduced affinity of binding ubiquitin^Phospho-Ser65^ (K_d_ of ∼42 μM) (right panel Fig3A and B). Overall, this indicates that His302 and Lys151 residues are required for optimal binding of ubiquitin^Phospho-Ser65^.

Table showing the *K*_d_ values (in μM), Δ*H* values (in kcal/mol), Δ*S* values (in kcal/mol deg) and −*T*Δ*S* (in kcal/mol) derived from the graphs. Data are representative of two independent experiments.

We also undertook complementary gel filtration analysis to assess for heterodimeric complex formation of wild-type Parkin with ubiquitin^Phospho-Ser65^. We exploited the above-described N-terminal Dac tag ubiquitin since this retains monomeric properties and enables visualisation of binding due to a large change in molecular weight that is easily visible by gel filtration analysis 24. We next produced a tagged fusion protein of Dac-ubiquitin^Phospho-Ser65^ or non-phosphorylated ubiquitin. Size-exclusion chromatography using a Superdex 200 Increase (10/300 GL) column was employed with 250 μl of purified protein at a concentration of 0.8 mg/ml. Incubation of Dac-ubiquitin with wild-type Parkin revealed no strong interaction and two separate peaks (FigEV3E). In contrast, we observed binding of wild-type Parkin with Dac-ubiquitin^PhosSer65^ with a shift in the size of the peaks indicating heterodimer formation (FigEV3F). We also observed binding of a Parkin S65A mutant with Dac-ubiquitin^PhosSer65^ (FigEV3K). Consistent with our ITC data, we observed no evidence of binding of the Parkin H302A mutant and Dac-ubiquitin^PhosSer65^ as judged by two separate peaks (FigEV3G).

**Figure:**
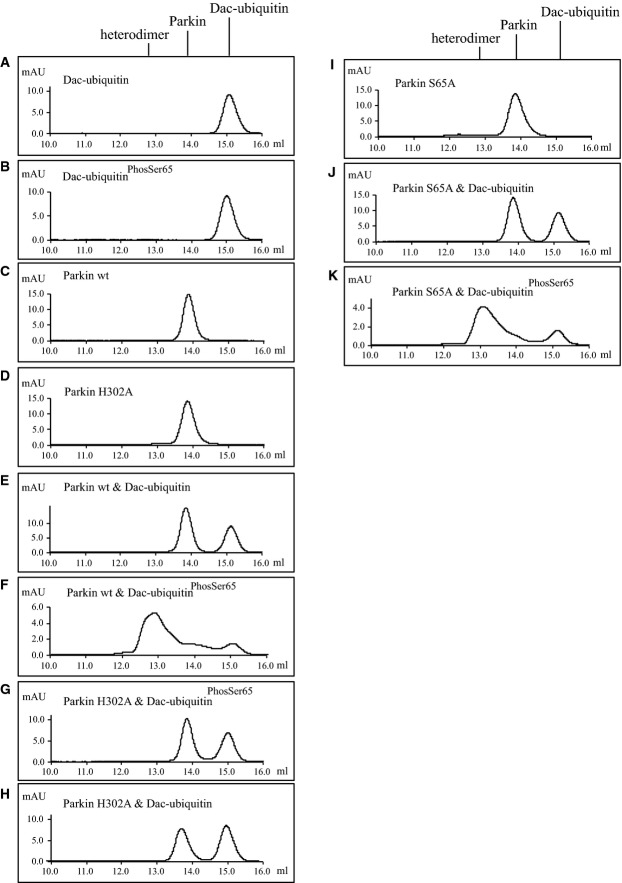
Analysis of heterodimer formation between Ser^65^-phosphorylated Dac-ubiquitin and Parkin using gel filtration chromatography About 100 μg of Dac-ubiquitin or Dac-ubiquitin^Phospho-Ser65^ (both ∼38.3 kDa) was incubated with or without 100 μg of wild-type Parkin (WT) or Parkin H302A (both ∼51.6 kDa) for 30 min and subjected to chromatography on a Superdex 200 Increase column (GE Healthcare Life Sciences). Parkin eluted at 13.8 ml and Dac-ubiquitin at 15.1 ml. The dimer eluted at 12.9 ml. The *x*-axis is millilitre elution volume, and the *y*-axis is arbitrary milli absorption units. Profile of Dac-ubiquitin.

Profile of Dac-ubiquitin^Phospho-Ser65^.

Profile of WT Parkin.

Profile of Parkin H302A mutant.

Profile of WT Parkin and Dac-ubiquitin.

Profile of WT Parkin and Dac-ubiquitin ^Phospho-Ser65^.

Profile of H302A Parkin and Dac-ubiquitin^Phospho-Ser65^.

Profile of H302A Parkin and Dac-ubiquitin.

Profile of S65A Parkin.

Profile of S65A Parkin and Dac-ubiquitin.

Profile of S65A Parkin and Dac-ubiquitin^Phospho-Ser65^.

Overall, our binding studies indicate a critical involvement of His302 and Lys151 towards ubiquitin^Phospho-Ser65^ binding and suggest that these residues may lie within the ubiquitin^Phospho-Ser65^ binding pocket of Parkin.

Whilst the His302 and Lys 151 residues are not mutated in human patients with Parkinson’s disease, a number of disease mutants lie in structural proximity including the RING1 mutant Arg275Trp (R275W) that we have previously shown to be inactive 26. We therefore investigated a panel of disease mutants including R275W that span all the domains of Parkin for their ability to exhibit enhancement of phosphorylation of Parkin Ubl Ser^65^ by PINK1 upon the addition of ubiquitin^Phospho-Ser65^. This resulted in the identification of three disease mutants K27N, A46P and G430D, of which K27N and A46P lie within the Ubl domain and G430D lies within the RING2 domain, that prevented the ability of ubiquitin^Phospho-Ser65^ to enhance Parkin Ser^65^ phosphorylation (FigEV4). However, since the basal phosphorylation of these proteins in the absence of ubiquitin^Phospho-Ser65^ was substantially lower than wild-type Parkin, it is possible that these mutants are trapped in a conformation in which the Ubl domain is less accessible. Elucidation of the crystal structure of these mutants in the full-length protein would test this hypothesis and particularly address how the RING2 mutant G430D that lies distant from the Ubl-RING1 interface could influence Ubl topology and Ser^65^ accessibility. Conversely, the R33Q mutant, which we previously found to be better phosphorylated than wild-type Parkin 26 due to presumed disruption of the Ubl domain autoinhibition, showed only a moderate change in phosphorylation by PINK1 upon addition of ubiquitin^Phospho-Ser65^ (FigEV4).

**Figure:**
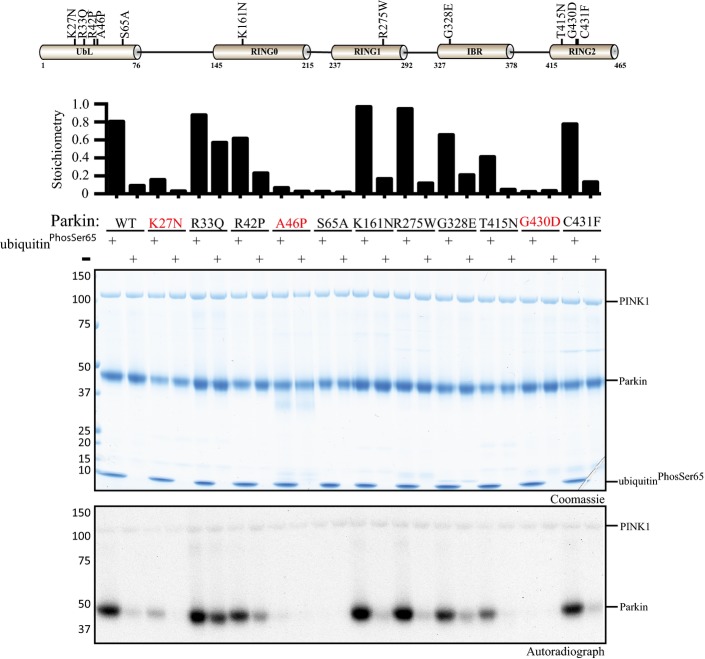
Identification of Parkinson’s disease-associated mutants that disrupt ubiquitin^Phospho-Ser65^-enhanced phosphorylation of Parkin by TcPINK1 Schematic of Parkin domain and location of disease-associated Parkin mutants (upper panel). Wild-type (WT) full-length Parkin or the indicated disease point mutant was incubated with wild-type MBP-TcPINK1 and Mg^2+^ [γ-^32^P] ATP in the presence or absence of ubiquitin^Phospho-Ser65^. Proteins were detected by Colloidal Coomassie Blue staining (top panel) and Parkin phosphorylation levels assessed by incorporation of [γ-^32^P] ATP detected by autoradiography (bottom panel) and displayed above the panel (lower Panel).

### Evidence that ubiquitin^Phospho-Ser65^ influences Parkin Ser^65^ phosphorylation in cells

**Figure 4:**
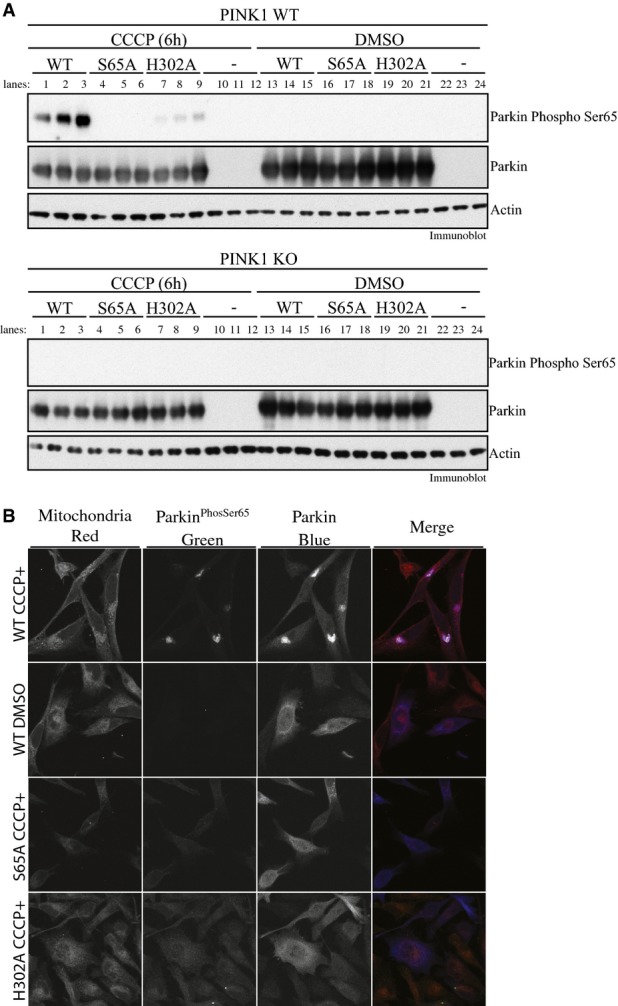
Parkin His302 is required for optimal phosphorylation of Parkin at Ser^65^ in cells upon CCCP-stimulated PINK1 activation Parkin H302A-mutant displays marked decrease in Parkin Ser^65^ phosphorylation upon PINK1 activation. Wild-type HeLa (upper panel) or PINK1 knockout HeLa cells (lower panel) were transfected with untagged wild-type (WT), and Ser65Ala (S65A)- or His302Ala-mutant Parkin and stimulated with 10 μM of CCCP or DMSO for 6 h in triplicates. The lysates were subjected to immunoblotting as follows: Parkin Ser^65^ phosphorylation (anti-phospho-Ser65 antibody), Parkin (anti-Parkin antibody), actin (anti-actin antibody) and PINK1 (anti-PINK1 antibody). Data are representative of three independent experiments.

We next addressed whether mutation of His302 influenced the ability of Parkin to become phosphorylated at Ser^65^ in cells upon activation of PINK1 kinase activity stimulated by CCCP-induced mitochondrial depolarisation. We expressed wild-type Parkin, a non-phosphorylatable S65A mutant and the H302A mutant of Parkin in both wild-type and PINK1 knockout HeLa cells 27. Cells were treated with 10 μM CCCP or DMSO for 6 h, and extracts were immunoblotted with a phospho-specific antibody raised against a previously characterised Parkin Ser^65^ epitope 9 (Fig11A). Consistent with our *in vitro* analysis (Fig2B), the level of Parkin phosphorylation was substantially diminished in cells expressing H302A Parkin (lanes 7–9, Fig4A) compared to wild-type Parkin (lanes 1–3, Fig4A). The specificity of the antibody was confirmed by the absence of significant phosphorylation in cells expressing S65A Parkin (lanes 4–6, Fig4A). The strict dependence of the Parkin Ser^65^ site on active PINK1 was also demonstrated by the lack of Parkin phosphorylation at Ser^65^ in PINK1 knockout cells. Under similar conditions, we also investigated whether His302 was critical for optimal Parkin E3 ligase activity in cells upon stimulation by mitochondrial depolarisation. Previous mass spectrometric analysis has identified numerous mitochondrial proteins as Parkin substrates including the Fe-S cluster-containing protein CISD1 25. Upon stimulation with CCCP, we observed a clear poly-ubiquitylation of CISD1 in mitochondrial extracts in cells expressing wild-type but not the catalytic inactive C431F RING2 mutant of Parkin (lane 1 and 4, Fig EV5). Strikingly, we observed a dramatic reduction in CISD1 poly-ubiquitylation in cells expressing mutant H302A (lane 3, FigEV5), suggesting that ubiquitin^Phospho-Ser65^ binding to His302 is essential for optimal Parkin activation in cells and is consistent with our *in vitro* analysis of the H302A mutant (Fig2B–D). We also observed a heavy reduction in CISD1 poly-ubiquitylation in cells expressing S65A, confirming the critical role of Ubl Ser^65^ phosphorylation in the activation of Parkin E3 ligase activity (lane 2, Fig EV5) in cells.

Parkin H302A-mutant disrupts mitochondrial accumulation of Parkin Ser^65^ phosphorylation. Wild-type HeLa cells stably expressing untagged wild-type (WT) (top row and second row), and Ser65Ala (S65A) (third row)- or His302Ala (H302A) (fourth row)-mutant Parkin were stimulated with 10 μM of CCCP for 6 h. HeLa cells expressing WT Parkin were also treated with DMSO for 6 h (second row). Cells were stained for Parkin Ser65 phosphorylation (anti-phospho-Ser65 antibody) or total Parkin (anti-Parkin antibody); mitochondria were labelled using MITO-ID® Red. Data representative of four independent experiments.

**Figure:**
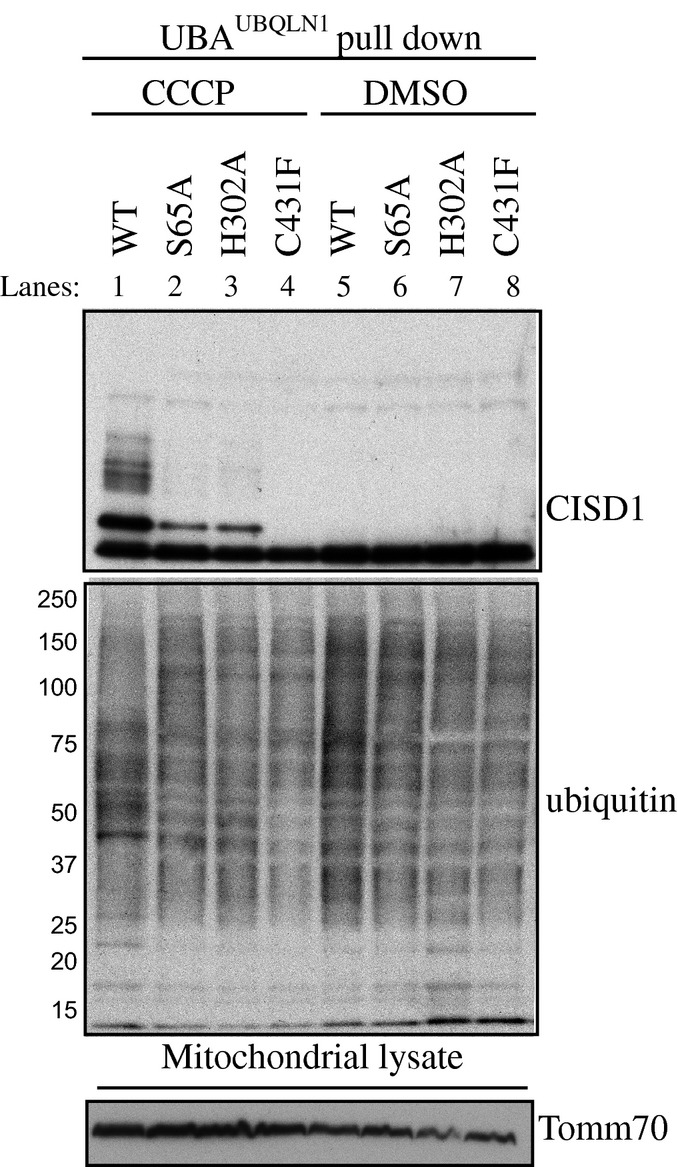
Parkin His302 is required for optimal activation of Parkin ubiquitin E3 ligase activity at mitochondria in response to PINK1 activation by CCCP Wild-type HeLa cells were transfected with untagged wild-type (WT), and Ser65Ala (S65A)-, His302Ala (H302A)- or Cys431Phe (C431F)-mutant Parkin and stimulated with 10 μM of CCCP or DMSO for 6 h. Mitochondrial enriched extracts were incubated with a ubiquitin-binding resin derived from His-Halo-Ubiquilin UBA-domain tetramer (UBA^UBQLN^^1^). Captured ubiquitylated proteins were subjected to immunoblotting with CISD1 and ubiquitin antibodies. In parallel, mitochondrial input extracts were immunoblotted with Tomm70 antibody.

In parallel, we also undertook immunofluorescence analysis of Parkin Ser^65^ phosphorylation in HeLa cells stably over-expressing wild-type and S65A- or H302A-mutant Parkin. Upon stimulation of cells expressing wild-type Parkin with 10 μM CCCP, we observed a striking accumulation of Parkin Ser^65^ phosphorylation on the mitochondria that had undergone peri-nuclear clustering as judged by staining with the mitochondrial dye Mito-ID Red (top row, Fig4B). As predicted with the high specificity of the antibody, we did not observe any Parkin Ser^65^ phosphorylation in cells expressing S65A Parkin following the treatment of CCCP (third row, Fig4B). Consistent with our Western blot analysis (Fig4A), we observed a large reduction of Parkin Ser^65^ phosphorylation in cells expressing H302A Parkin. Interestingly, the Ser^65^-phosphorylated H302A Parkin was confined mainly to the cytosol (fourth row, Fig4B).

Overall, our cell-based analysis supports the notion that ubiquitin^Phospho-Ser65^ binding to Parkin via His302 is required for optimal phosphorylation of Parkin Ser^65^ by PINK1 in cells. The diffuse Parkin H302A Ser^65^ signal in the cytosol is consistent with Parkin accumulation to mitochondria being dependent on binding to ubiquitin^Phospho-Ser65^ 21,28 and suggests a role for ubiquitin^Phospho-Ser65^ in regulating Parkin retention on the depolarised mitochondria. In future studies, it would be interesting to undertake time–course analysis of Parkin phosphorylation and ubiquitylation activity at the mitochondria for wild-type Parkin and the H302A mutant. This may distinguish between the possibilities of whether H302A is initially phosphorylated on the mitochondria but cannot be retained over time and whether there is no Parkin Ubl Ser^65^ phosphorylation at all on mitochondria due to its inability to bind ubiquitin^Phospho-Ser65^ in the first instance.

### Ubiquitin^Phospho-Ser65^ disrupts Ubl binding to Parkin

We next investigated the mechanism by which ubiquitin^Phospho-Ser65^ enhances PINK1-dependent phosphorylation of Parkin Ubl Ser^65^. The Ubl domain binds to the RING1 domain 12 and can also interact with Parkin lacking the Ubl domain *in trans* 11. Crystal and NMR solution structures of the Ubl domain reveal that Ser^65^ resides in a loop adjacent to the fifth β-strand that exhibits conformational flexibility 29-31 and may only be partially surface accessible in the full-length structure. We therefore hypothesised that ubiquitin^Phospho-Ser65^ binding might disrupt the ability of the Ubl domain to bind to the C-terminal domain and therefore render Ser^65^ of the Ubl domain more accessible for phosphorylation by PINK1.

**Figure 5:**
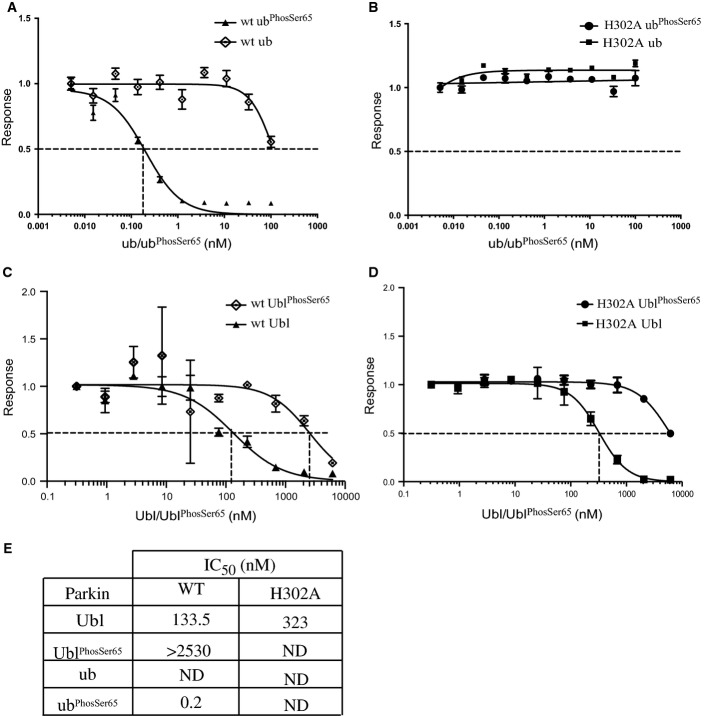
Ubiquitin^Phospho-Ser65^-mediated disruption of the Ubl domain with ΔUbl Parkin is dependent on residue His302 An AlphaScreen™ binding assay of Parkin Ubl domain and C-terminus of Parkin were established. GST-Ubl (residues 1-76) and wild-type C-terminal Parkin-biotin (residues 80-465) were incubated with streptavidin-coated donor beads and glutathione acceptor beads for 60 min. A, B Ubiquitin^Phospho-Ser65^ (ub^PhosSer65^) disrupts maximal binding signal of Parkin and Ubl interaction of wild-type (wt) (A), but not H302A (H302A) Parkin (B).

To address this question, we developed an AlphaScreen® *in vitro* binding assay to quantify the interaction between Parkin Ubl domain (GST-Ubl, residues 1-76) and a C-terminal fragment of Parkin that contains the RING1 domain (Parkin (80-465)-Biotin) (Fig5; Appendix Fig S4). Streptavidin-coated donor beads were used in conjunction with glutathione-coated acceptor beads, and the binding was established by monitoring proximity-based luminescent signal. The optimal binding conditions were determined by titrating GST-Ubl (1-76) 0–300 nM against Parkin (80-465)-biotin 0–300 nM. Cross-titration analysis revealed optimal binding with concentrations of 10 nM Parkin (80–465)-biotin and 10 nM GST-Ubl (1-76). Strikingly, we observed that addition of increasing amounts of ubiquitin^Phospho-Ser65^ but not non-phospho-ubiquitin led to dose-dependent disruption of GST-Ubl (1-76) binding to Parkin (80-465)-biotin with an IC_50_ of ∼0.2 nM (Fig5A and E). This is consistent with the notion that binding of Parkin to ubiquitin^Phospho-Ser65^ disrupts the interaction of the Ubl domain with the C-terminal domain. We next assessed the role of the His302 residue in the ubiquitin^Phospho-Ser65^-mediated disruption of Ubl binding. We observed that the GST-Ubl to Parkin H302A-mutant binding was insensitive to increasing amounts of ubiquitin^Phospho-Ser65^ (Fig5B and E) consistent with the His302 residue playing an essential role in binding ubiquitin^Phospho-Ser65^ and promoting PINK1 phosphorylation and activation of Parkin (Figs2 and 3). Consistent with previous stability analysis of full-length Parkin H302A mutant (Fig EV2A and B), the E3 ligase activity of Parkin H302A (80-465)-biotin was similar to wild-type Parkin (FigEV2B).

C, D Ubl^Phospho-Ser65^ (Ubl^PhosSer65^) exhibits substantially reduced ability to disrupt interaction between GST-Ubl and wild-type (C) and H302A Parkin (D).

E Table showing the IC_50_ values (in nM) derived from graphs, where the interaction inhibition is incomplete, and not-determined (ND) is indicated. Data information: Data show mean of one trial ± s.d., *n* = 3 for each condition (A–D).

To assess the interplay between ubiquitin^Phospho-Ser65^- and the Ser^65^-phosphorylated Ubl domain (Ubl^Phospho-Ser65^) moieties in the activation of Parkin, we undertook AlphaScreen assays using WT and H302A Parkin and investigated the ability of Ubl^Phospho-Ser65^ to disrupt these interactions. Under similar assay conditions, we observed that the ability of Ubl^Phospho-Ser65^ to disrupt the GST-Ubl to Parkin WT interaction was greatly reduced compared to ubiquitin^Phospho-Ser65^ and the observed mild effect was likely to be mediated by the presence of unphosphorylated Ubl (< 10%) present in the Ubl^Phospho-Ser65^ sample (Fig5C and E). This is consistent with the lack of the effect of Ubl^Phospho-Ser65^ in promoting PINK1-dependent phosphorylation of Parkin (Fig1C). Similarly, Ubl^Phospho-Ser65^ was only able to weakly disrupt the GST-Ubl to Parkin H302A interaction (Fig5D and E) that is again likely to be due to the effect of unphosphorylated Ubl in the Ubl^Phospho-Ser65^ sample. The elaboration of this AlphaScreen® binding assay also has the potential to identify small molecules that act as Parkin activators by mimicking the effect of ubiquitin^Phospho-Ser65^.

### Purified Ser^65^–phosphorylated Parkin exhibits constitutive activity that is no longer sensitive to ubiquitin^Phospho-Ser65^

Our data indicate that binding of ubiquitin^Phospho-Ser65^ to Parkin may represent a critical first step of the Parkin activation cascade. This suggests that the ensuing Parkin Ubl Ser^65^ phosphorylation by PINK1 is likely to represent the critical effector step leading to the activation of Parkin E3 ligase activity. To obtain further experimental evidence for this model, we investigated whether near-maximally phosphorylated purified Ser^65^-phosphorylated Parkin (Parkin^Phospho-Ser65^) could be further activated by the addition of ubiquitin^Phospho-Ser65^. His-SUMO-tagged full-length Parkin was initially captured on Ni-NTA-agarose, phosphorylated by MBP-TcPINK1, before the His-SUMO tag was removed and Parkin was purified via nickel affinity and size exclusion chromatography. Under super-saturating phosphorylation conditions, we were able to purify a heterogeneous protein population, wherein approximately 60% Parkin was phosphorylated as confirmed directly by AQUA peptide analysis (Appendix Fig S5).

**Figure 6:**
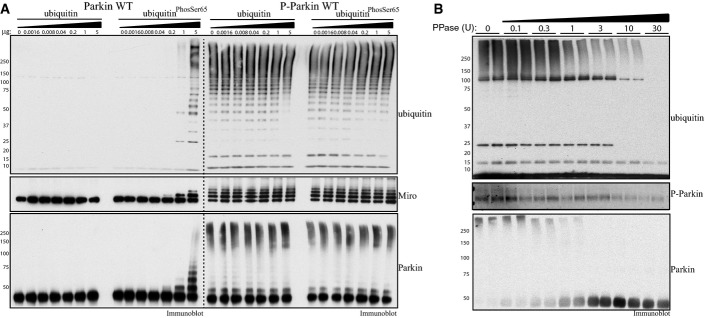
Purified Parkin phosphorylated at Ser65 exhibits constitutive E3 ligase activity that is no longer sensitive to ubiquitin^Phospho-Ser65^ Parkin phosphorylated at Ser65 exhibits significant constitutive activity. About 2 μg of full-length wild-type (Parkin WT) or phosphorylated at Ser65 (P-Parkin WT) Parkin was analysed using E3 ligase assay with increasing amounts of non-phospho-ubiquitin or ubiquitin^Phospho-Ser65^ as indicated. Parkin activity was evaluated by immunoblotting as follows: ubiquitin (anti-FLAG-HRP antibody), Parkin (anti-Parkin antibody) and Miro1 (anti-SUMO1 antibody). The molecular mass in kDa is indicated. Data are representative of three independent experiments.

We compared the activity of Parkin^Phospho-Ser65^ with that of wild-type Parkin in the presence or absence of increasing amounts of non-phosphorylated ubiquitin or ubiquitin^Phospho-Ser65^. As expected, the purified Parkin^Phospho-Ser65^ exhibited marked E3 ligase activity in the absence of ubiquitin^Phospho-Ser65^ as judged by free poly-ubiquitin chain formation, Miro1 multi-monoubiquitylation and Parkin autoubiquitylation (Fig6A). This activity was substantially greater than the activity observed for wild-type Parkin as evident by Miro1 ubiquitylation. Most importantly, we observed no further enhancement of the activity of Parkin^Phospho-Ser65^ upon the addition of molar excess amounts of ubiquitin^Phospho-Ser65^ that led to maximal activation of full-length wild-type Parkin (Fig6A). This was not likely the result of the Parkin^Phospho-Ser65^ activity being at saturated levels since no change was observed when ubiquitin^Phospho-Ser65^ was added to a 10-fold Parkin^Phospho-Ser65^ dilution that displayed markedly reduced constitutive E3 ligase activity (Appendix Fig S6). Furthermore, we undertook time–course analysis of Parkin E3 ligase activity and continued to observe no enhancement of Parkin^Phospho-Ser65^ activity at 5 min in which the E3 ligase activity of Parkin^Phospho-Ser65^ had not reached saturation as judged by free poly-ubiquitin chain formation and Parkin autoubiquitylation (Appendix Fig S7). We also generated ∼90% phosphorylated H302A-mutant Parkin (Appendix Fig S5) and observed that this was as active as wild-type Parkin^Phospho-Ser65^ (Appendix Fig S8), providing further evidence that the H302A mutation is not significantly perturbing the structural fold and integrity of Parkin. To further confirm that the constitutive activity of Parkin^Phospho-Ser65^ was indeed due to phosphorylation, we treated Parkin^Phospho-Ser65^ with increasing amounts of calf alkaline phosphatase and observed a dose-dependent reduction in its activity as judged by free poly-ubiquitin chain formation and Parkin autoubiquitylation that correlated with decreased phosphorylation at Parkin Ser^65^ (Fig6B).

Dephosphorylation of Parkin at Ser65 leads to reversal of Parkin E3 ligase constitutive activity. About 1 μg of full-length Parkin phosphorylated at Ser65 was subjected to increasing amounts of alkaline phosphatase as indicated. Reactions were then incubated with ubiquitylation assay components (E1 and UbcH7) in the presence of 0.05 mM FLAG-ubiquitin. Reactions were terminated after 60 min by the addition of LDS loading buffer. The effects on Parkin E3 ligase activity were evaluated by ubiquitin chain formation ubiquitylation and Parkin autoubiquitylation evaluated by immunoblotting as follows: ubiquitin (anti-FLAG-HRP antibody) and Parkin (anti-Parkin antibody). The degree of Parkin Ser^65^ de-phosphorylation was monitored using anti-pSer65 Parkin antibody. Data are representative of four independent experiments.

Further structural analysis will be needed to better understand how ubiquitin^Phospho-Ser65^ mediates Parkin activation. Currently, it has been shown that His302 residue lies within the RING1 domain distal from the Ubl domain 12-14. Elucidation of the co-complex of ubiquitin^Phospho-Ser65^ bound to Parkin in an activated conformation should define all the key residues on both Parkin and ubiquitin^Phospho-Ser65^ that are critical for binding and define the molecular role of the phospho-Ser^65^-binding pocket in mediating Parkin activation.

## Conclusions

**Figure 7:**
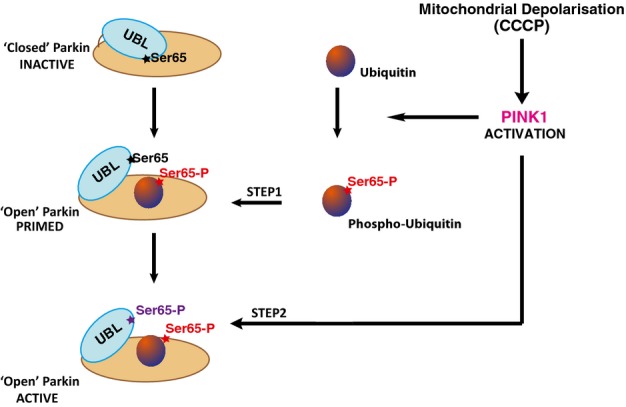
Priming model of Parkin activation by PINK1-dependent phosphorylation of ubiquitin Upon activation of PINK1 by mitochondrial depolarisation, PINK1 can phosphorylate ubiquitin to generate phospho-ubiquitin^Phospho-Ser65^. Binding of ubiquitin^Phospho-Ser65^ to non-phosphorylated Parkin can disrupt intramolecular interaction of Ubiquitin-like (Ubl) domain to Parkin C-terminus. The Ser65 residue on Ubl becomes more accessible for PINK1-dependent phosphorylation leading to an open and active conformation of Parkin.

Recent models of Parkin activation by PINK1 have highlighted a role for ubiquitin^Phospho-Ser65^ in binding and recruiting phosphorylated Parkin to mitochondria triggering a feed-forward amplification loop of Parkin activity at the mitochondria 19-28. The data presented here demonstrate that another major role of ubiquitin^Phospho-Ser65^ is to prime Parkin Ubl Ser^65^ for phosphorylation by PINK1. Furthermore, we have found that once phosphorylated, Parkin^Phospho-Ser65^ is no longer sensitive to ubiquitin^Phospho-Ser65^, suggesting that the E3 ligase activity of Parkin may largely be driven by PINK1-mediated phosphorylation of the Ubl domain of Parkin at Ser^65^ that may stabilise it in the active conformation. Our findings elaborate a 2-step model of Parkin activation by ubiquitin^Phospho-Ser65^ and the PINK1 kinase (Fig7) that offers novel insights into how Parkin is activated and adds another layer of interdependence between Parkin Ubl^Phospho-Ser65^ and ubiquitin^Phospho-Ser65^ that would be predicted to drive feed-forward amplification of Parkin E3 ligase activity. Our findings not only provide fundamental insights into the regulation of Parkin but also have implications for designing strategies to search for small molecule activators of Parkin.

## Materials and Methods

### Materials

[γ-^32^P] ATP was from Perkin Elmer. HaloLink resin was purchased from Promega. All mutagenesis was carried out using the QuikChange site-directed mutagenesis method (Stratagene) with KOD polymerase (Novagen). All DNA constructs were verified by DNA sequencing, which was performed by The Sequencing Service, School of Life Sciences, University of Dundee, using DYEnamic ET terminator chemistry (Amersham Biosciences) on Applied Biosystems automated DNA sequencers. DNA for bacterial protein expression was transformed into *E. coli* BL21 DE3 RIL (codon plus) cells (Stratagene). All cDNA plasmids, antibodies and recombinant proteins generated for this study are available to request through our reagents website (https://mrcppureagents.dundee.ac.uk/).

### Antibodies

Antigen affinity-purified sheep anti-SUMO-1 antibody was a kind gift from Professor Ron Hay (Dundee) (IB: 1:2,000). Anti-Parkin mouse monoclonal was obtained from Santa Cruz (sc-32282) (IB: 1:5,000; IF: 1:1,000); anti-FLAG HRP (A8592) (IB: 1:10,000) and anti-actin (A2066) (IB: 1:5,000) antibodies were obtained from Sigma; anti-CISD1 (16006-1-AP) (IB: 1:1,000) and anti-TOMM70A (14528-1-AP) (IB: 1:1,000) antibodies were obtained from Proteintech Europe; and anti-ubiquitin antibody (Z0458) (IB: 1:1,000) was purchased from Dako. Epitomics raised anti-Parkin phospho-serine 65 rabbit monoclonal antibody in collaboration with the Michael J Fox Foundation for Research (IB: 1:1,000; IF: 1:500).

### Cell culture

HeLa PINK1 knockout cell lines were obtained from Richard Youle (NIH) and were cultured using DMEM (Dulbecco’s modified Eagle’s medium) supplemented with 10% FBS (foetal bovine serum), 2 mM L-glutamine, 1× Pen/Strep and 1× non-essential amino acids (Life Technologies). Flp-In T-Rex HeLa stable cell lines were cultured in the above-mentioned media supplemented with 15 μg/ml of blasticidin and 100 μg/ml of hygromycin. Cell transfections of untagged Parkin were performed using polyethylene method 32. To uncouple mitochondria, cells were treated with 10 μM CCCP (Sigma) dissolved in DMSO for 6 h.

### Membrane fraction enrichment

Cells were collected in ice-cold PBS containing 200 mM chloroacetamide. They were then lysed in buffer containing 250 mM sucrose, 20 mM HEPES, 3 mM EDTA, 1% (w/v) 1 mM sodium orthovanadate, 10 mM sodium β-glycerophosphate, 50 mM NaF, 5 mM sodium pyrophosphate, pH 7.5 and protease inhibitor cocktail (Roche) supplemented with 100 mM chloroacetamide at 4°C. Cells were disrupted using a glass hand-held homogeniser (40 passes), and the lysate was clarified by centrifuging for 10 min at 800 *g* at 4°C. The supernatant was further centrifuged at 16,600 *g* for 10 min. The pellet containing the mitochondrial fraction was resuspended in buffer containing 1% Triton X-100 and centrifuged at 13,000 r.p.m. for 10 min. This supernatant contained solubilised mitochondrial proteins.

### Ubiquitin enrichment in membrane fractions

His-Halo-Ubiquilin UBA-domain tetramer (UBA^UBQLN1^) was expressed in *E.coli* BL21 cells, affinity purified on Ni-NTA-agarose and dialysed into 50 mM HEPES pH 7.5, 10% glycerol, 150 mM NaCl and 1 mM DTT. UBA^UBQLN1^ was coupled to HaloLink Resin as described in 23. About 1 mg of mitochondria-enriched fractions was then subjected to pull-downs as described in 23.

### Immunoblotting

Samples were subjected to SDS–PAGE (4–12%) and transferred on to nitrocellulose membranes. Membranes were blocked for 1 h in Tris-buffered saline with 0.1% Tween (TBST) containing 5% (w/v) non-fat dried skimmed milk powder. Membranes were probed with the indicated antibodies in TBST containing 5% (w/v) non-fat dried skimmed milk powder overnight at 4°C. Detection was performed using HRP-conjugated secondary antibodies and enhanced chemiluminescence reagent.

### Immunofluorescence

HeLa cells stably expressing untagged Parkin WT, S65A or H302A were plated on glass coverslips and treated as described. Immunofluorescence was performed as described in 33. Briefly, coverslips were washed twice with phosphate-buffered saline (PBS), fixed with 3.7% formaldehyde, 50 mM HEPES pH 7.0 for 10 min, washed twice with and then incubated for 10 min with DMEM, 10 mM HEPES pH 7.4. Cells were permeabilised by incubation with 0.2% Triton X-100 in PBS followed by two washes and blocking for 15 min at RT with PBS supplemented with 1% BSA (PBS/1% BSA). Cells were stained with the primary antibodies as follows: Parkin^Phospho-Ser65^ (1:500) antibody for 16 h at 4°C and total Parkin antibody (1:1,000) for 1 h at 37°C, followed by anti-mouse or anti-rabbit Alexa Fluor 405- or Alexa Fluor 488-conjugated secondary antibodies (Life Technologies). The mitochondria was stained using MITO-ID® Red detection kit (Enzo Life Sciences) at for 30 min at 37°C.

Immunofluorescently labelled cells were imaged using the Zeiss LSM 700 laser scanning confocal microscope with the Alpha Plan-Apochromat ×100/NA 1.46 objective (optical section thickness 0.7 μm). Parkin-Alexa 405 was excited with the 405 laser, P-Parkin-Alexa 488 was excited with the 488 laser, and Mitochondria MITO-ID® Red was excited with the 555 laser. All labels were excited independently to prevent cross-channel bleed through.

### Analysis of Ser^65^ phosphorylation of Parkin by AQUA peptides and mass spectrometry

In order to quantify phosphorylation stoichiometry, 2 ng of Parkin or phospho-Parkin was digested with trypsin and analysed by absolute quantitation selected reaction monitoring (SRM) LC mass spectrometry 34. Samples were separated by a 60-min gradient on a 50-cm Acclaim PepMap 100 analytical column (75 μm ID, 3 μm C18) in conjunction with a PepMap trapping column (100 μm × 2 cm, 5 μm C18) (Thermo Fisher Scientific) in a Dionex Ultimate 3000 Nano LC system (Dionex/Thermo Fisher) coupled to a QTRAP 5500 (ABSCIEX) mass spectrometer. Synthetic peptides were used to define retention time and the optimal 10 transitions per peptide, based on optimal intensity of precursor charge state and fragment ions. One pmol of heavy labelled peptides of Parkin NDWTVQN[C(CAM)]DLDQQSIVHIVQRPW[**R**(13C6; 15N4)], R.NDWTVQN[C(CAM)]DLDQQ[S(PO3H2)]IVHIVQRPW**R**(13C6; 15N4)] (AQUA QuantPro, Thermo Scientific) was mixed with the trypsin digest of phospho-Parkin, and the ratio of peak intensities of ten distinct transitions for light (endogenous) and heavy peptides was used to calculate the amount of endogenous proteins. Data were analysed with Skyline software 35.

### *In vitro* ubiquitylation assays

Wild-type Parkin^Phospho-Ser65^ or indicated mutant of Parkin (2 μg or 0.2 μg) was incubated with ubiquitylation assay components in a final volume of 50 μl (50 mM Tris–HCl (pH 7.5), 5 mM MgCl_2_, 0.12 μM UbE1, 1 μM UbcH7 and 2 μg 6xHis-Sumo-Miro, 2 mM ATP). About 5 μg of ubiquitin or ubiquitin^Phospho-Ser65^ was added as indicated. Ubiquitylation reactions were incubated at 30°C for 60 min and terminated by the addition of LDS sample buffer. For all assays, reaction mixtures were resolved by SDS–PAGE. Ubiquitylation reactions were subjected to immunoblotting with anti-FLAG antibody (Sigma, 1:10,000), anti-Parkin (Santa Cruz 1:5,000) or anti-SUMO1 (1:2,000).

### *In vitro* E2 discharge assays

E2-charging reaction was assembled in 5 μl containing Ube1 (0.5 μg), an UbcH7 (2 μg), 50 mM HEPES pH 7.5 and 10 μM ubiquitin in the presence of 2 mM magnesium acetate and 0.2 mM ATP. After initial incubation of 60 min at 30°C, the reactions were combined with 2 μg of WT or indicated mutant of Parkin in the presence or absence of 1 μg of ubiquitin or ubiquitin^Phospho-Ser65^ and allowed to continue for a further 15 min at 30°C. Reactions were terminated by the addition of 5 μl of LDS loading buffer and subjected to SDS–PAGE analysis in the absence of any reducing agent. Gels were stained using InstantBlue.

### Kinase assays

Reactions were set up in a volume of 25 μl, using 2 μg of WT or indicated mutants of Parkin and 1 μg of wild-type or kinase-inactive (D359A) MBP-TcPINK1, in 50 mM Tris–HCl (pH 7.5), 0.1 mM EGTA, 10 mM MgCl_2_, 0.1% β-mercaptoethanol and 0.1 mM [γ-^32^P] ATP. About 1 μg of different ubiquitin species or indicated amounts of WT, S65A or ubiquitin^Phospho-Ser65^ was added as indicated. Assays were incubated at 30°C and terminated after 60 min by the addition of SDS sample loading buffer. The reaction mixtures were then resolved by SDS–PAGE. Proteins were detected by Coomassie staining, and gels were imaged using an Epson scanner and dried completely using a gel dryer (Bio-Rad). Incorporation of [γ-^32^P] ATP into substrates was analysed by autoradiography using Amersham Hyper-Film.

### Buffers for *E. coli* protein purification

For Parkin purification, lysis buffer contained 50 mM Tris–HCl (pH 7.5), 150 mM NaCl, 1 mM EDTA, 1 mM EGTA, 5% (v/v) glycerol, 1% (v/v) Triton X-100, 0.1% (v/v) 2-mercaptoethanol, 1 mM benzamidine and 0.1 mM PMSF. Wash buffer contained 50 mM Tris–HCl (pH 7.5), 500 mM NaCl, 0.1 mM EGTA, 5% (v/v) glycerol, 0.03% (v/v) Brij-35, 0.1% (v/v) 2-mercaptoethanol, 1 mM benzamidine and 0.1 mM PMSF. Equilibration buffer contained 50 mM Tris–HCl (pH 7.5), 150 mM NaCl, 0.1 mM EGTA, 5% (v/v) glycerol, 0.03% (v/v) Brij-35, 0.1% (v/v) 2-mercaptoethanol, 1 mM benzamidine and 0.1 mM PMSF. Elution buffer was equilibration buffer with the addition of 12 mM maltose. Storage buffer was equilibration buffer with the addition of 0.27 M sucrose and glycerol—PMSF and benzamidine were omitted.

### Protein purification from *E. coli*

Full-length, wild-type and kinase-inactive TcPINK1 was expressed in *E. coli* as maltose-binding protein (MBP) fusion protein and purified as described previously 36. Briefly, BL21 CodonPlus-transformed cells were grown at 37°C to an OD_600_ of 0.3, then shifted to 16°C and induced with 250 μM IPTG (isopropyl β-D-thiogalactoside) at OD_600_ of 0.5. Cells were induced with 250 μM IPTG at OD 0.6 and were further grown at 16°C for 16 h. Cells were pelleted at 4,000 rpm and then lysed by sonication in lysis buffer. Lysates were clarified by centrifugation at 30,000 *g* for 30 min at 4°C followed by incubation with 1 ml per litre of culture of amylose resin for 1.5 h at 4°C. The resin was washed thoroughly in wash buffer and then equilibration buffer, and proteins were then eluted. Proteins were dialysed overnight at 4°C into storage buffer, snap-frozen and stored at −80°C until use.

Wild-type and indicated mutant untagged Parkin (His-SUMO cleaved) was expressed and purified using a modified protocol as previously described 18.

Ser^65^-phosphorylated Parkin was produced by the expression of His-SUMO-tagged Parkin and then captured by Ni^2+^-NTA-Sepharose as described above. After extensive washes, captured His-SUMO-Parkin was incubated with MBP-PINK (Parkin: PINK1 ratio of 2:1) twice consecutively for 3 h in the presence of 0.5 mM ATP and 10 mM Mg-acetate at 27°C. PINK1 was removed, and Parkin was eluted with 0.4 M imidazole and further incubated for 16 h with MBP-PINK1 in solution. The proteins were concentrated using Vivaspin filters and then diluted again to reduce the imidazole concentration to 20 mM to recapture His-SUMO-Parkin on Ni-agarose. Residual MBP-PINK1 was removed by extensive washes, before His-SUMO-Parkin was eluted with 0.4 M imidazole and further purified as described above.

Wild-type 6xHis-Sumo-Miro1 (1–592) was expressed in *E. coli*. Briefly, BL21 CodonPlus (DES)-RIL-transformed cells were grown at 37°C to an OD_600_ of 0.4, then reduced to 15°C and induced with 10 μM IPTG at an OD_600_ of 0.6. Cells were then grown at 15°C for a further 20 h. Cells were pelleted at 4,200 *g* and then lysed by sonication in lysis buffer. Lysates were clarified by centrifugation at 30,000 *g* for 30 min at 4°C followed by incubation with cobalt resin at 4°C for 45 min. The resin was washed thoroughly in high salt buffer and equilibrated in low salt buffer, and the proteins were then eluted. The eluted Miro1 proteins were further purified by anion exchange chromatography. Proteins were applied to a Mono-Q HR 5/5 column and chromatographed with a linear gradient of NaCl from 0 M to 0.5 M. Fractions containing the purified Miro1 protein were then dialysed, snap-frozen in liquid nitrogen and stored at −70°C.

### Purification of Ser^65^-phosphorylated ubiquitin and Ser^65^-phosphorylated Parkin Ubl domain (residues 1-76)

About 23 μM bovine ubiquitin (SIGMA) was phosphorylated for 24 h with 3.7 μM MBP-PINK1 at 22°C in the presence of 100 μM ATP and 10 mM MgCl_2_. To replace ADP with ATP, the reaction was dialysed against Mg-ATP solution. Ubiquitin was filtered through a 30-kDa Vivaspin filter to remove MBP-PINK1, concentrated in a 3-kDa MWCO filter device, washed extensively with water and loaded onto a Mono-Q column, which did not bind ubiquitin, but phospho-ubiquitin. The latter was recovered by washing the column with 50 mM Tris pH 7.5, which was sufficient to elute stoichiometrically phosphorylated ubiquitin. Similarly, Parkin Ubl domain (residues 1-76) was expressed as previously described 9 and was phosphorylated with MBP-TcPINK1, recovered by filtration and applied to a Mono-Q column. Phospho-Parkin Ubl bound to the column and eluted with about 100 mM NaCl. At least, 90% purity was achieved.

### Purification of Dac-ubiquitin and Ser^65^-phosphorylated Dac-ubiquitin

The Dac tag is a fragment of *E. coli* penicillin-binding Protein 5, comprising residues 37-297, where the N-terminus was modified to MSAIPG to allow efficient mRNA translation 24. pET28-Dac-ubiquitin was transformed into BL21 cells. Protein expression was induced with 250 μM IPTG for 16 h at 26°C. Cells were sedimented and lysed in 50 mM Tris pH 7.5, 0.5% Triton X-100, 0.1 mM EDTA, 0.1 mM EGTA, 1 mM Pefabloc® and 10 μg/ml leupeptin. After sonication, the insoluble material was removed by centrifugation. Dac-ubiquitin fusion protein was captured by incubation for 45 min at 22°C with ampicillin-Sepharose. The ampicillin-Sepharose was thoroughly washed, and the protein was eluted with 50 mM Tris pH 7.5, 150 mM NaCl, 5% glycerol, 10 mM ampicillin and 0.03% Brij35. The protein was dialysed into 40 mM HEPES, pH 7.5, 100 mM NaCl and 1 mM DTT.

Ser^65^-phosphorylated Dac-ubiquitin **(**Dac-ubiquitin^PhosSer65^) was prepared by phosphorylation of Dac-ubiquitin with GST-*Pediculus humanus* PINK1 126-end (ratio Dac-Ub: GST PhPINK of 6.25:1) for 3 h in the presence of 20 mM Mg-acetate and 2 mM ATP. Dac-ubiquitin was repurified over ampicillin-Sepharose and concentrated to match the concentration of Dac-ubiquitin.

### Gel filtration chromatography analysis of complexes

A Superdex 200 Increase 10/300 GL column (GE Healthcare Life Sciences) was equilibrated with 50 mM Tris pH 7.5 and 150 mM NaCl. The proteins: 100 μg of Dac-ubiquitin, Dac-ubiquitin^PhosSer65^, Parkin (1-465), Parkin 1-465 S65A, or Parkin 1-465 H302A or mixtures thereof, were made and subjected to chromatography on the column at a flowrate of 0.3 ml/min. Mixtures were incubated for 30 min prior to chromatography. The elution of the proteins was monitored by UV absorption at 280 nm.

### MALDI analysis

MALDI-TOF was used to confirm and establish the ratios of phosphorylated versus non-phosphorylated protein species. An aliquot of the reaction (2 μl, 400–600 fmols) was added to 2 μl of the matrix (2,5-dihydroxyacetophenone, 15 mg/ml in 80% ethanol, 20% of 12 mg/ml ammonium citrate bibasic), and 2 μl of 2% (v/v) trifluoroacetic acid was added before spotting 0.5 μl of the sample on to an AnchorChip target (Bruker Daltonics). The analysis was performed manually in linear positive mode using an UltrafleXtreme (Bruker Daltonics) MALDI–TOF mass spectrometer. For external calibration, six average masses were used: insulin [M+H]^+^ avg (m/z 5734.520), cytochrome c [M+2H]^2+^ avg (m/z 6181.050), myoglobin [M+2H]^2+^ avg (m/z 8476.660), ubiquitin I [M+H]^+^ avg (m/z 8565.760) and cytochrome C [M+H]^+^ avg (m/z 12360.970).

### AlphaScreen binding assay

Serial three-fold, 10-point dilution curves of inhibitor proteins ubiquitin^PhosSer65^ and ubiquitin were prepared in PBS + 0.1% BSA + 0.1% Triton X-100 with a maximum concentration of 400 nM. Similar three-fold 10-point dilution curves of Ubl^PhosSer65^ and Ubl were produced with a maximum concentration of 24.7 mM. Biotinylated wild-type or H302A-mutant Parkin protein and GST-Ubl were diluted to 40 nM in PBS + 0.1% BSA +0.1% Triton X-100. 5 μl of inhibitor, 5 μl of GST-Ubl and 5 μl of Parkin protein were added to a white-walled 384-well plate (Griener Bio-one). Plates were centrifuged at 500 *g* for 3 min and mixed on an orbital shaker at 450 rpm for 3 min prior to incubation at RT for 20 min. About 5 μl of a 20 μg/ml mixture of streptavidin donor and glutathione acceptor beads in PBS + 0.1% BSA + 0.1% Triton X-100 was added to all wells. Plates were briefly centrifuged and incubated at RT in the dark for 1 h prior to reading on PHERAstar (BMG LABTECH) using an AlphaLISA optical module (excitation wavelength = 680 nm and emission wavelength = 615 nm).

### Isothermal calorimetric assay

ITC measurements were carried out on an ITC_200_ Microcalorimeter (GE Healthcare). Wild-type, and His302Ala- and Lys151Ala-mutant Parkin and ubiquitin^PhosSer65^ were dialysed in buffer containing 50 mM HEPES (pH 8.0), 150 mM NaCl and 500 μM of TCEP. The sample cell containing 50 μM of wild-type or mutant Parkin and sufficient amount of ubiquitin^PhosSer65^ was titrated in the injection syringe to achieve a complete binding isotherm. All binding experiments were undertaken in duplicate at constant temperature of 20°C. A total of 20 injections of 2.0 μl were dispensed with a 5-s addition time and spacing of 120 s. Data were analysed and titration curves fitted using MicroCal Origin software assuming a single binding site mode.

### Thermal shift assay

Thermal denaturation experiments were performed using *Differential Scanning Fluorimetry* (DSF). 5 μg of wild-type, and His302Ala- or Lys151Ala-mutant Parkin protein was added to 45 μl reaction buffer (50 mM Hepes pH 8.0, 150 mM NaCl, 500 μM TCEP) and 2.5 μl of 100× Sypro Orange (Invitrogen) fluorescent dye to yield a final reaction volume of 50 μl. Each experiment was repeated three times in a 96-well plate in a Bio-Rad iQ5 thermal cycler, with a temperature gradient set from 10°C to 95°C at steps of 0.5°C per min.

## Supporting Information

**Appendix**

**Expanded View Figures PDF**

**Review Process File**
