# Supplementary material for: Binding to serine 65-phosphorylated ubiquitin primes Parkin for optimal PINK1-dependent phosphorylation and activation
Source: EMBO Rep. 2015 Jun 26;16(8):939–54. doi: 10.15252/embr.201540352 (PMC4552487; doi:10.15252/embr.201540352)

Appendix File:

Appendix Figure Legends

Appendix Figures S1 – S8

Appendix Figure Legends

Appendix Figure S1. Ubiquitin^{PhosphoSer65} specifically promotes Parkin Ser^{65} phosphorylation by PINK1. The effect of ubiquitin^{PhosphoSer65} on the ability of TcPINK1 to phosphorylate wild-type (WT), Ser65Ala (S65A), and Ubl-deleted (ΔUbl; residues 80-465) Parkin was investigated in a kinase assay. The indicated Parkin species was incubated with or without ubiquitin^{PhosphoSer65}in a kinase assay analysed by SDS/PAGE. Proteins were detected by Colloidal Coomassie Blue staining (top panel) and incorporation of [γ-^{32}P] ATP was detected by autoradiography (bottom panel).

Appendix Figure S2. The mitochondrial protein HAX1 is phosphorylated by TcPINK1 *in vitro*. GST-HAX1 (full length) (2 μM) was incubated in the presence of full-length wild-type TcPINK1 (1–570) and kinase inactive TcPINK1 (D359A) and Mg2+ [γ-^{32}P] ATP. Assays were terminated by addition of LDS loading buffer and separated by SDS-PAGE. Proteins were detected by Colloidal Coomassie Blue staining (upper panel) and incorporation of [γ-^{32}P] ATP was detected by autoradiography (lower panel).

Appendix Figure S3. Parkin His302 and Lys151 are highly conserved across multiple species. Sequence alignment of residues around Lys151 and His302 in human Parkin and a variety of lower organisms showing high degree of conservation.

Appendix Figure S4. Expression of proteins utilised in Alphascreen binding assay. 2μg of the following proteins were analysed by SDS/PAGE and proteins were detected by Colloidal Coomassie Blue. Parkin Ubl (residues 1n 76), GSTn Parkin Ubl, Parkin (80n 465), Parkin (80n 465)n biotin affinity peptide (BAP), Parkin (80n 465)n biotin.

Appendix Figure S5. Quantitative analysis of phosphorylated full length Parkin at Ser^{65} using Aquapeptides for p-Ser^{65}. Table summarising the peptides in wild-type Parkin (WT) (left), WT Parkin^{PhosphoSer65} (P-Parkin WT) samples (middle), and H302A Parkin^{PhosphoSer65} (P-Parkin H302A) samples (right). LC-MS/MS spectra (left panel) of non-phospho peptides of WT Parkin (top), P-Parkin WT (middle) and P-Parkin H302A (bottom). LC-MS/MS spectra (right panel) of phospho peptides of WT Parkin (top), P-Parkin WT (middle) and P-Parkin H302A (bottom).

Appendix Figure S6. Low titre of phosphorylated Parkin at Ser^{65} activity remains insensitive to ubiquitin^{PhosphoSer65}. 0.22 μg of wild-type (Parkin WT) or Parkin phosphorylated at Ser65 (P-Parkin WT) were incubated in an E3 ligase

assay supplemented with non-phospho-ubiquitin or ubiquitin^{PhosphoSer65} as indicated. Parkin activity was evaluated by immunoblotting as follows: ubiquitin (anti-FLAG-HRP antibody; 1:10000) and Miro-1 (anti-SUMO1 antibody; 1:2000).

Appendix Figure S7. Timecourse analysis of phosphorylated Parkin at Ser^{65} activity in the presence or absence of ubiquitin^{PhosphoSer65}. 2 μg of wild-type (Parkin WT) or Parkin phosphorylated at Ser65 (P-Parkin WT) were subjected to E3 ligase assays in the presence of non-phospho-ubiquitin or ubiquitin^{PhosphoSer65} as indicated. Reactions were terminated at the indicated timepoints by the addition of LDS loading buffer. Parkin activity was evaluated by immunoblotting as follows: ubiquitin (anti-FLAG-HRP antibody; Sigma, 1:10000), and Parkin (anti-Parkin antibody; Santa Cruz, 1:5000).

Appendix Figure S8. Purified phosphorylated His302Ala Parkin at Ser^{65} exhibits constitutive E3 ligase activity that is insensitive to ubiquitin^{PhosphoSer65}. 2 μg of full-length wild-type (P-Parkin WT) or His302Ala (P-Parkin H302A) Parkin phosphorylated at Ser65 were subjected to E3 ligase assays supplemented with non-phospho-ubiquitin or ubiquitin^{PhosphoSer65} as indicated. Parkin activity was evaluated immunoblotting as follows: ubiquitin (anti-FLAG-HRP antibody; Sigma, 1:10000), Parkin (anti-Parkin antibody; Santa Cruz, 1:5000) and Miro-1 (anti-SUMO1 antibody; 1:2000).

Appendix Figure S1

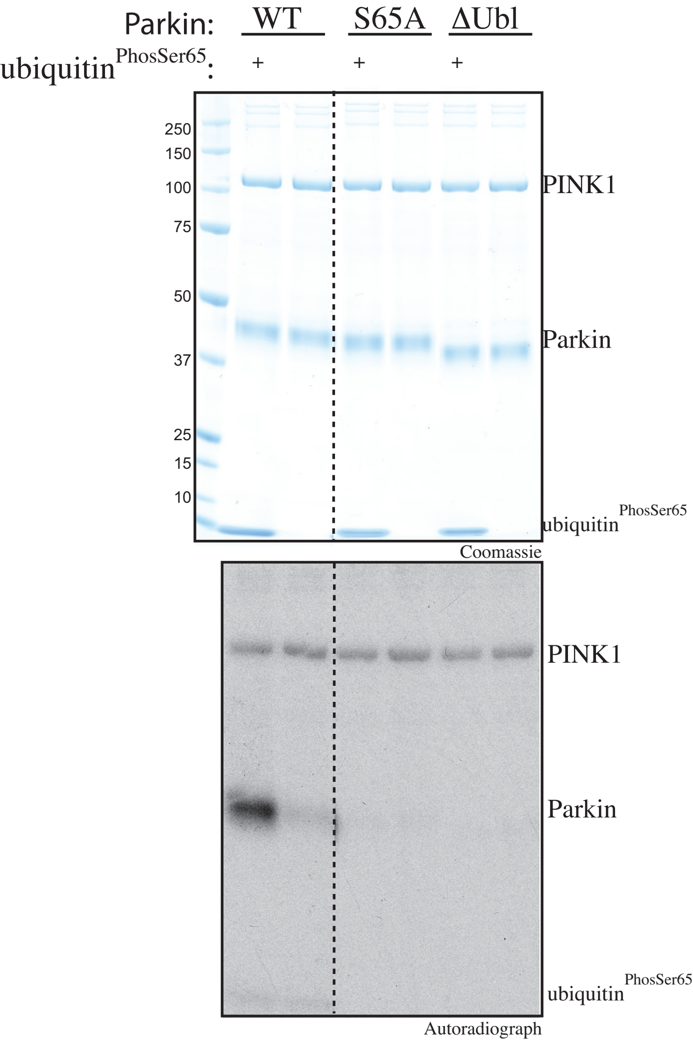

Appendix Figure S2

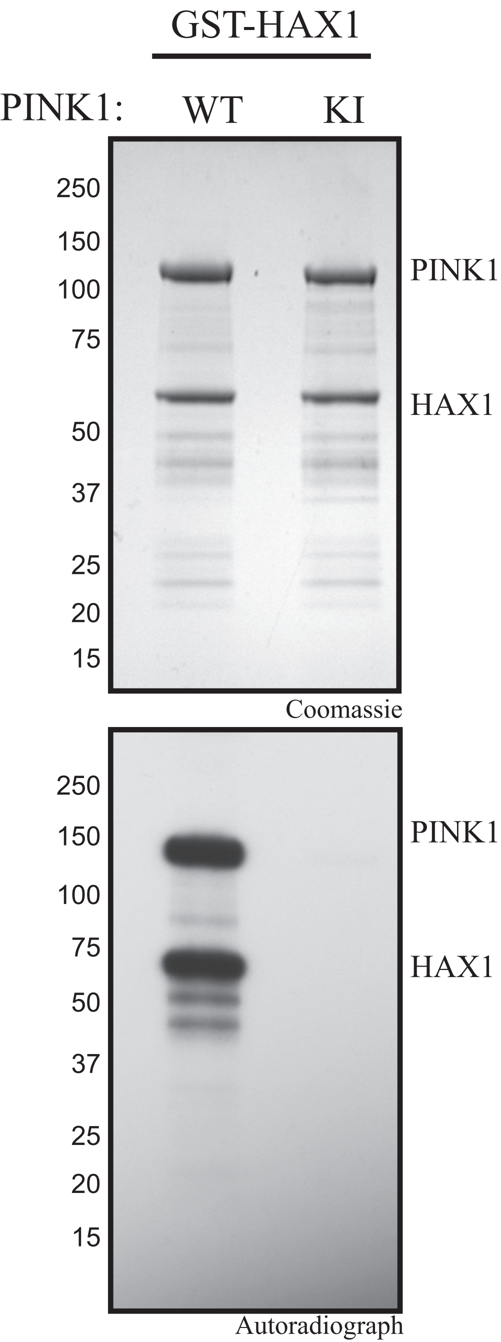

Appendix Figure S3

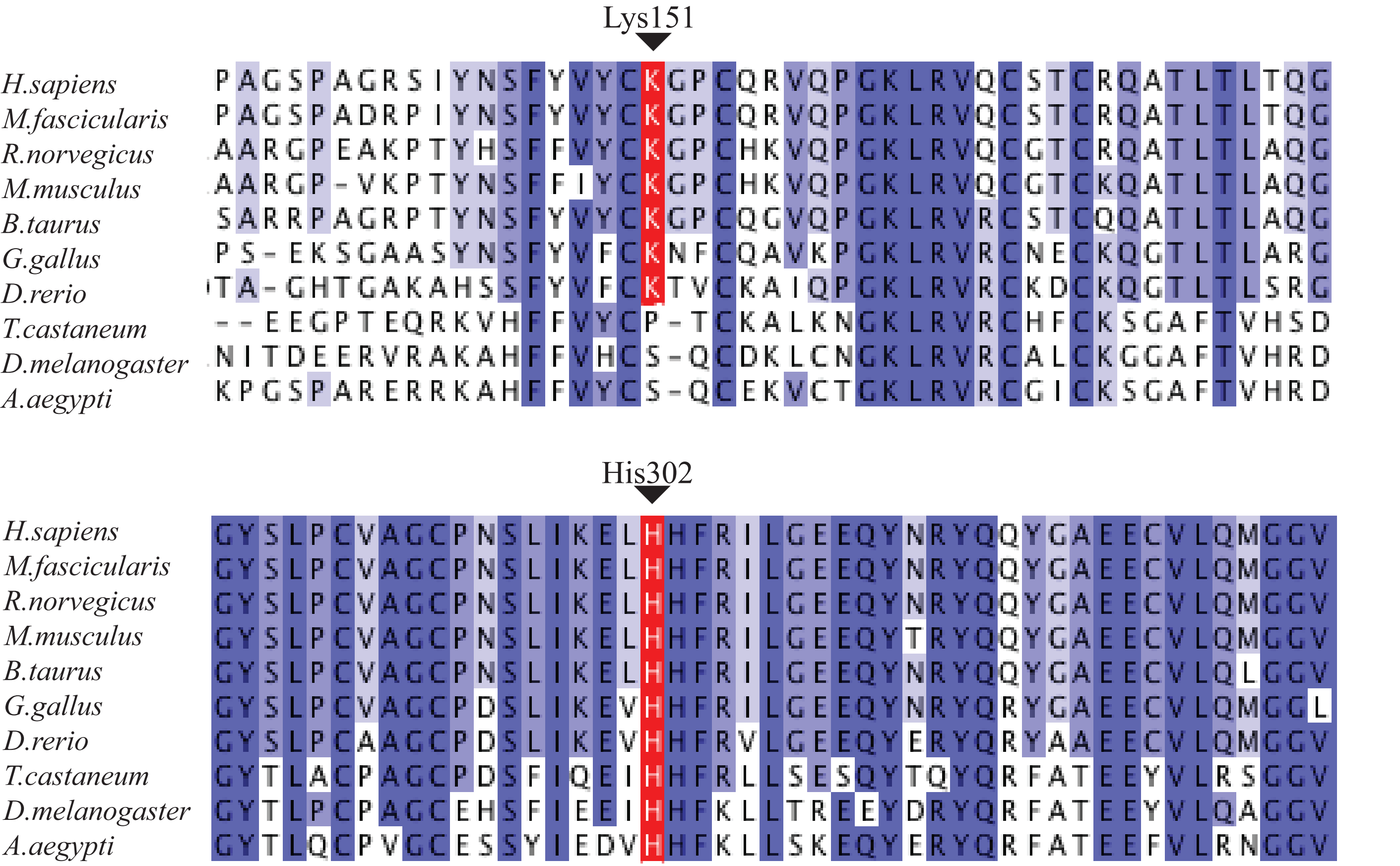

Appendix Figure S4

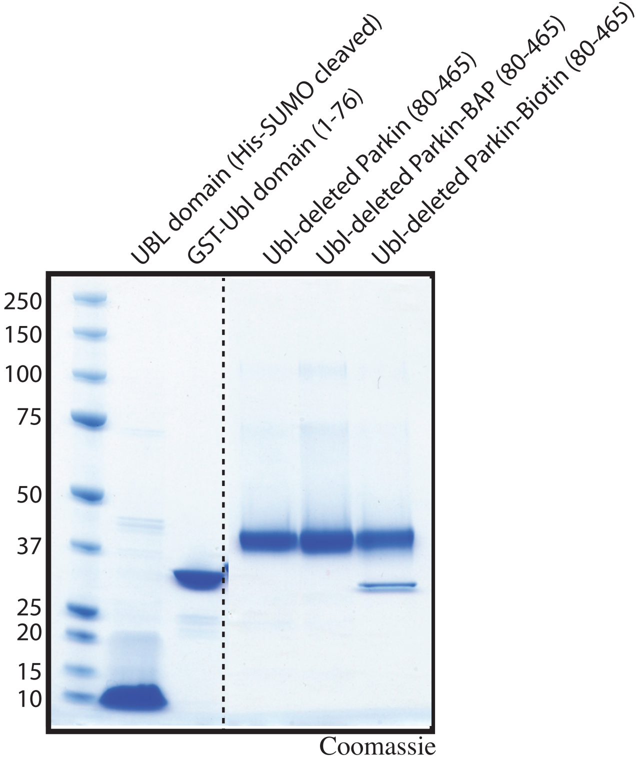

Appendix Figure S5

	Parkin WT	p-Parkin WT	p-Parkin H302A
non-phosphorylated (%)	91	40	6
phosphorylated (%)	background	60	94

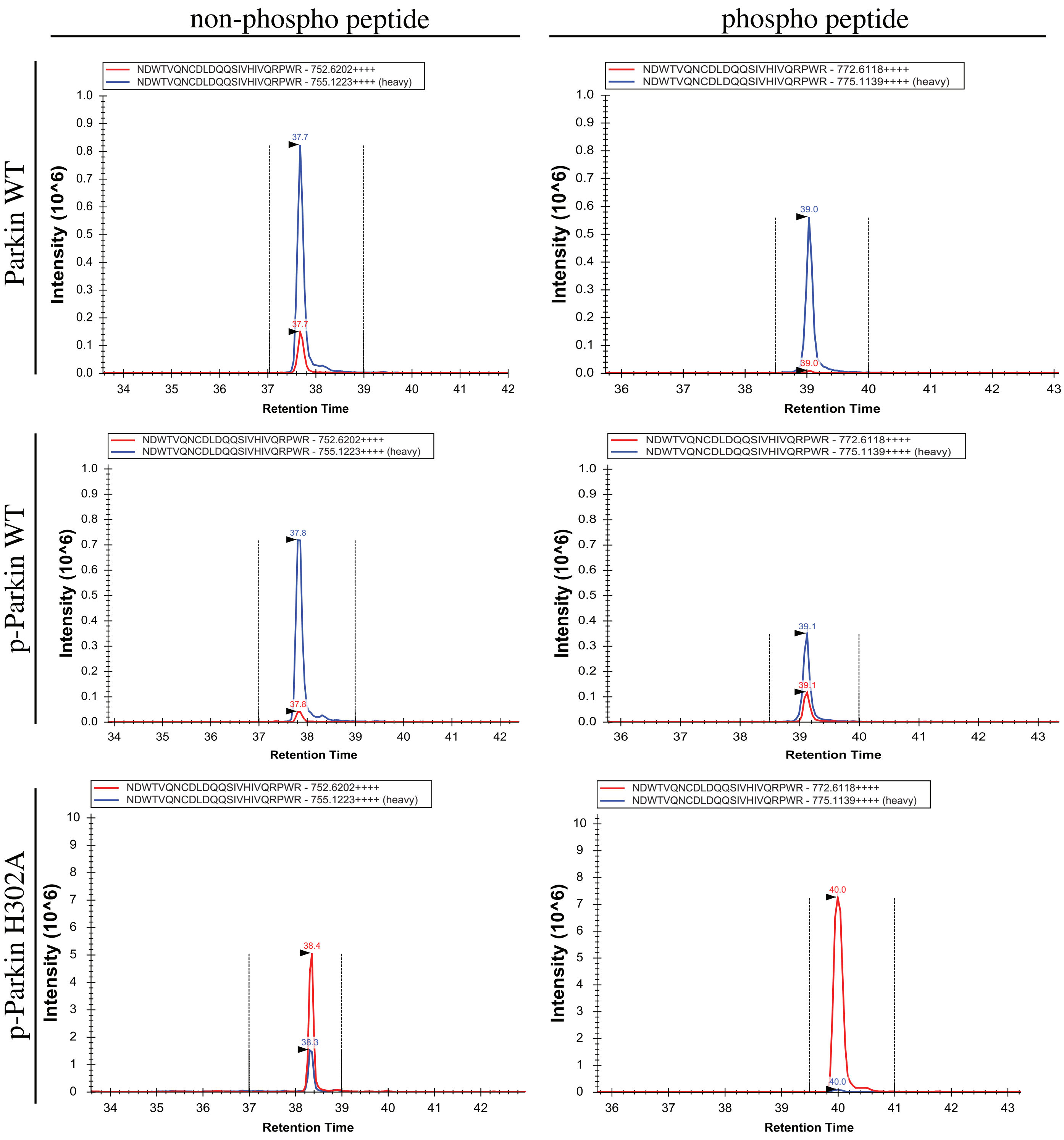

Appendix Figure S6

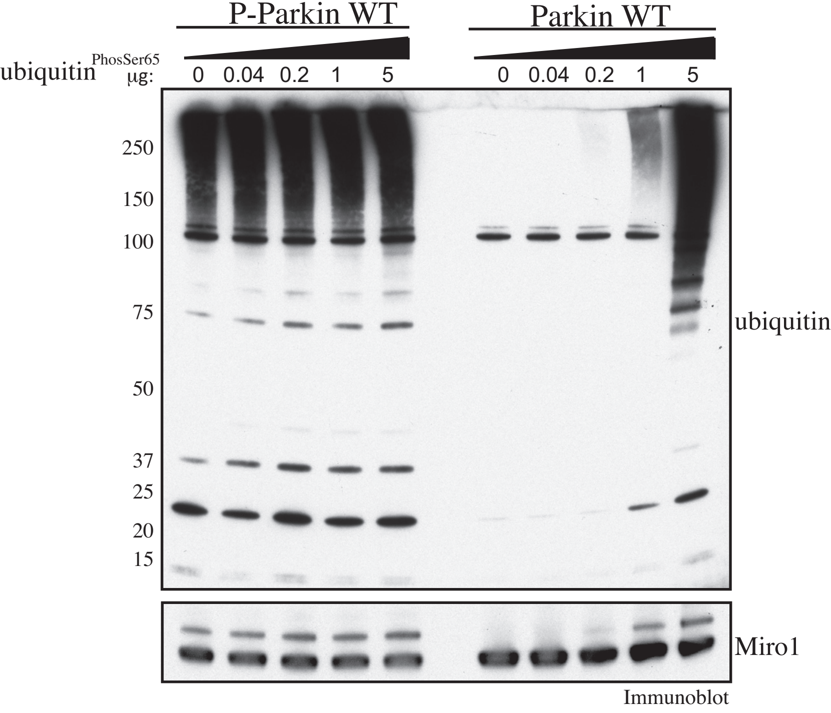

Appendix Figure S7

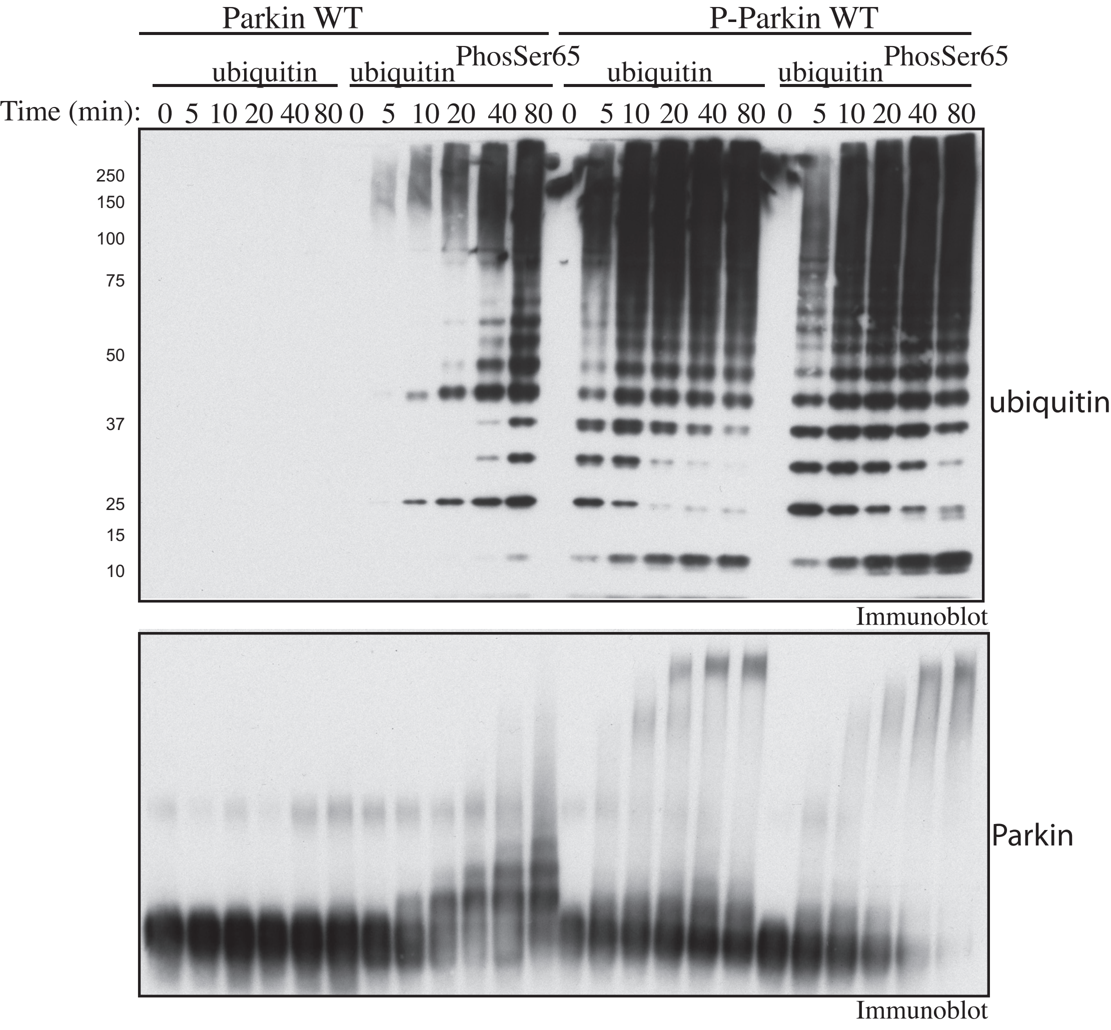

Appendix Figure S8

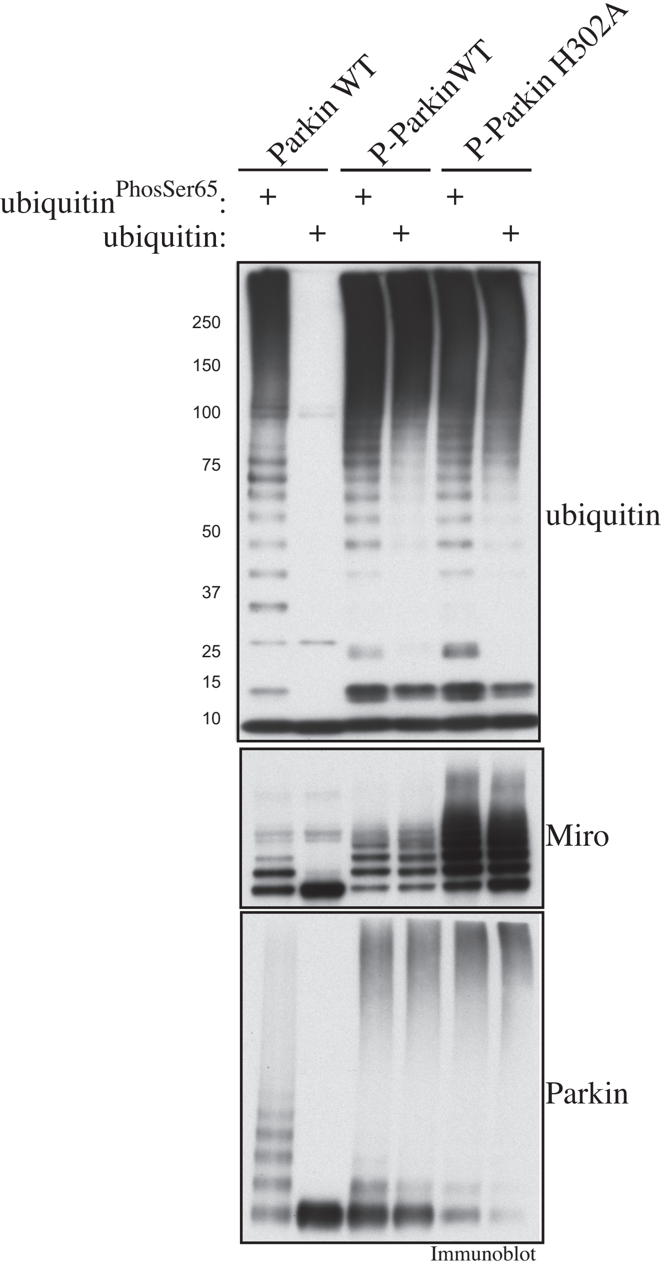

Supplement: Supplementary file 1 [file embr0016-0939-sd1.pdf]
