# Supplementary material for: Binding to serine 65-phosphorylated ubiquitin primes Parkin for optimal PINK1-dependent phosphorylation and activation
Source: EMBO Rep. 2015 Jun 26;16(8):939–54. doi: 10.15252/embr.201540352 (PMC4552487; doi:10.15252/embr.201540352)
Supplement: Supplementary file 2 [file embr0016-0939-sd2.pdf]

Expanded View Figures

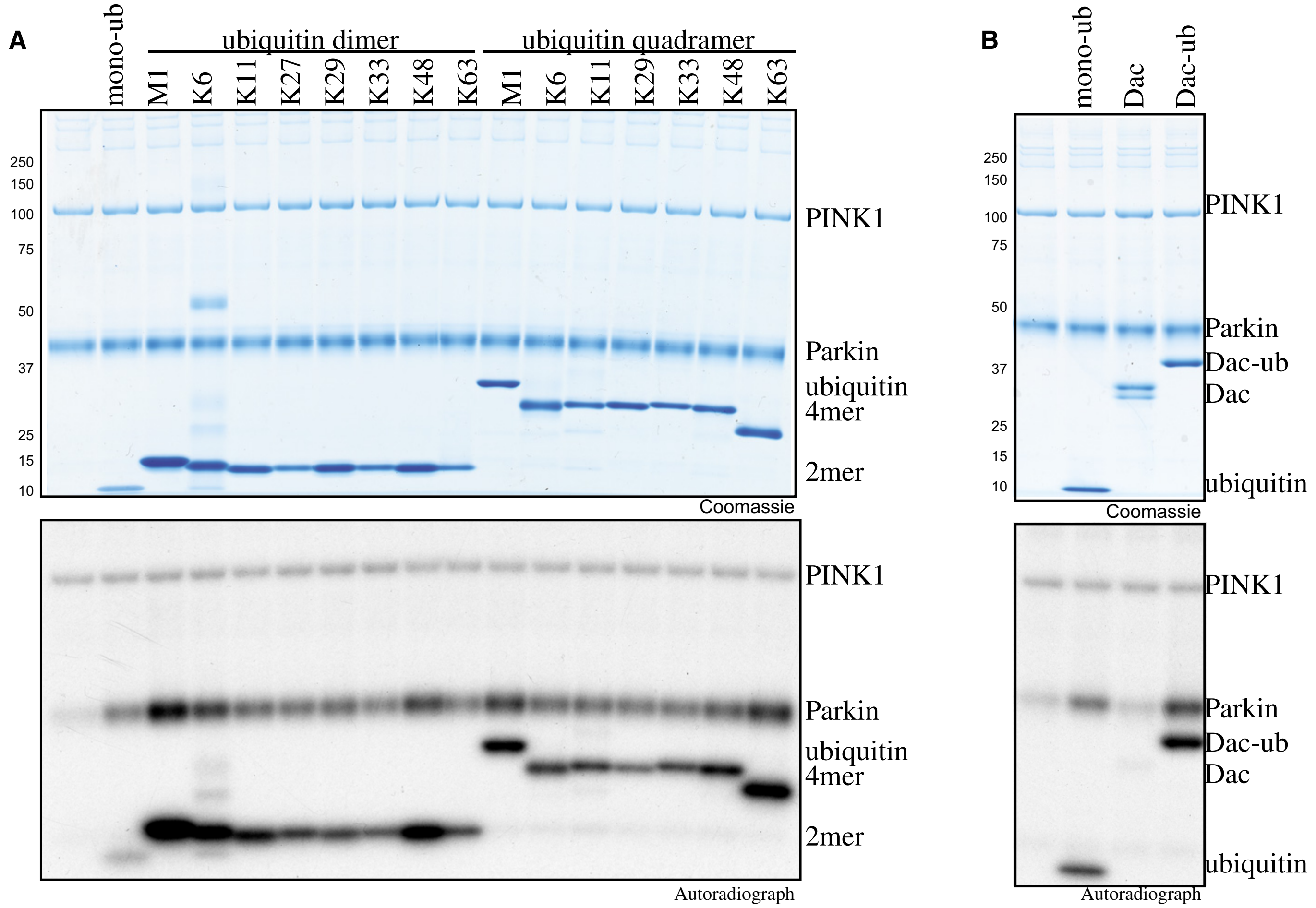

Figure EV1. Ubiquitin dimers, tetramers or a mono-ubiquitylated substrate are capable of priming Parkin phosphorylation by PINK1.

A, B (A) The effects of ubiquitin dimers of each linkage type (Met1 (M1), Lys6 (K6), Lys11 (K11), Lys27 (K27), Lys 29 (K29), Lys33 (K33), Lys48 (K48) and Lys63 (K63), ubiquitin tetramers with M1, K6, K11, K29, K33, K48 and K63 linkages and (B) the model mono-ubiquitylated substrate, Dac-ubiquitin (Dac-ub) were assessed in a PINK1 kinase assay similar to that in Fig 1. Assays were analysed by SDS/PAGE. Proteins were detected by Colloidal Coomassie Blue staining (top panel), and incorporation of [γ-^{32}P] ATP was detected by autoradiography (bottom panel).

© 2015 The Authors

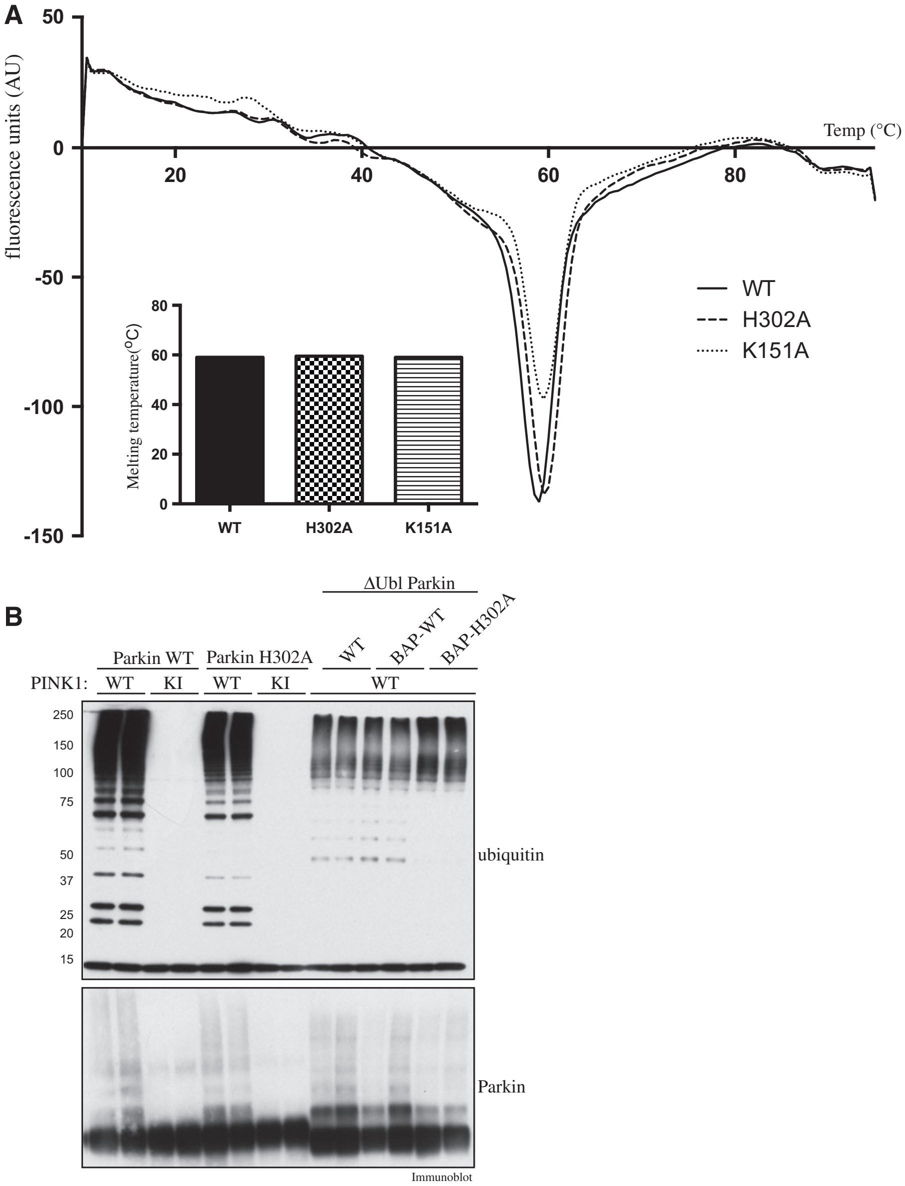

Figure EV2. Analysis of stability and ubiquitin$^{Phospho\text{-}Ser65}$-independent E3 ligase activity of "Pocket 2" mutants of Parkin.

A Thermal denaturation curves obtained by differential scanning fluorimetry of wild-type (WT), and His302Ala (H302A)- and Lys151Ala (K151A)-mutant Parkin. Results are displayed as the differential of the fluorescence in arbitrary units divided by the differential of the temperature, plotted against temperature. Inset: the minimum of each curve indicates the melting point (Tm). Summary of melting points (Tm) of each protein as follows: WT (59°C); H302A (59.5°C); and K151A (59.5°C).

B PINK1-dependent full-length Parkin E3 ligase activity mediated via phosphorylation of Ubl Ser65 and constitutive basal E3 ligase activity mediated by Ubl-deleted Parkin (ΔUbl; residues 80-465) are not affected by His302Ala (H302A) mutation. A 2 μg amount of wild-type full-length or ΔUbl-Parkin-biotin and H302A full-length or ΔUbl-Parkin was incubated with 1 μg of wild-type (WT), kinase-inactive (KI) or no TcPINK1 in an E3 ligase assay. Reactions were terminated after 60 min by the addition of LDS loading buffer and analysed by SDS/PAGE. Ubiquitin and Parkin were detected using anti-FLAG and anti-Parkin antibodies, respectively.

 © 2015 The Authors

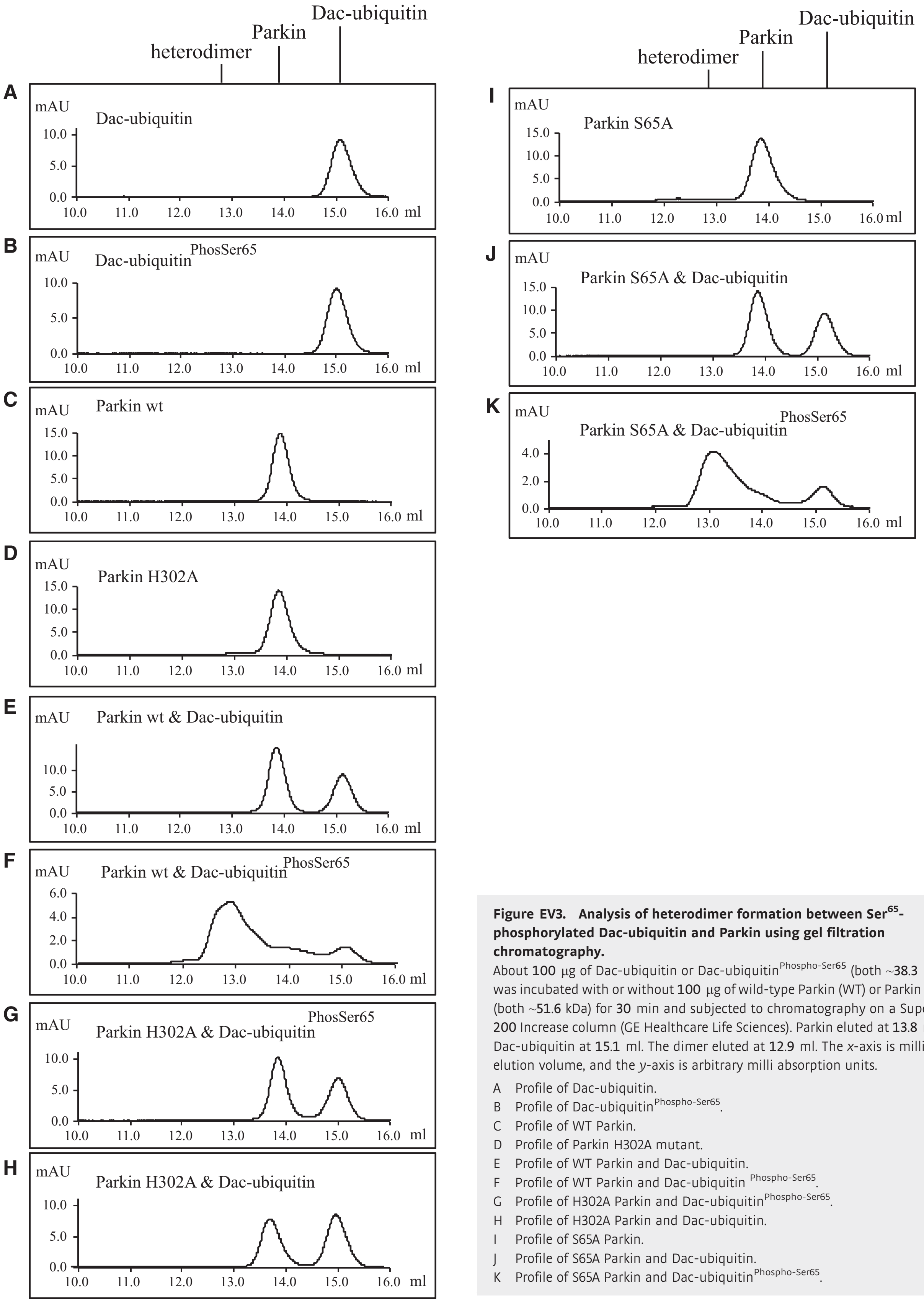

Figure EV3. Analysis of heterodimer formation between Ser[65]-phosphorylated Dac-ubiquitin and Parkin using gel filtration chromatography.

About 100 μg of Dac-ubiquitin or Dac-ubiquitin$^{\text{Phospho-Ser65}}$ (both ~38.3 kDa) was incubated with or without 100 μg of wild-type Parkin (WT) or Parkin H302A (both ~51.6 kDa) for 30 min and subjected to chromatography on a Superdex 200 Increase column (GE Healthcare Life Sciences). Parkin eluted at 13.8 ml and Dac-ubiquitin at 15.1 ml. The dimer eluted at 12.9 ml. The *x*-axis is millilitre elution volume, and the *y*-axis is arbitrary milli absorption units.

A Profile of Dac-ubiquitin.
B Profile of Dac-ubiquitin$^{\text{Phospho-Ser65}}$.
C Profile of WT Parkin.
D Profile of Parkin H302A mutant.
E Profile of WT Parkin and Dac-ubiquitin.
F Profile of WT Parkin and Dac-ubiquitin $^{\text{Phospho-Ser65}}$.
G Profile of H302A Parkin and Dac-ubiquitin$^{\text{Phospho-Ser65}}$.
H Profile of H302A Parkin and Dac-ubiquitin.
I Profile of S65A Parkin.
J Profile of S65A Parkin and Dac-ubiquitin.
K Profile of S65A Parkin and Dac-ubiquitin$^{\text{Phospho-Ser65}}$.

© 2015 The Authors

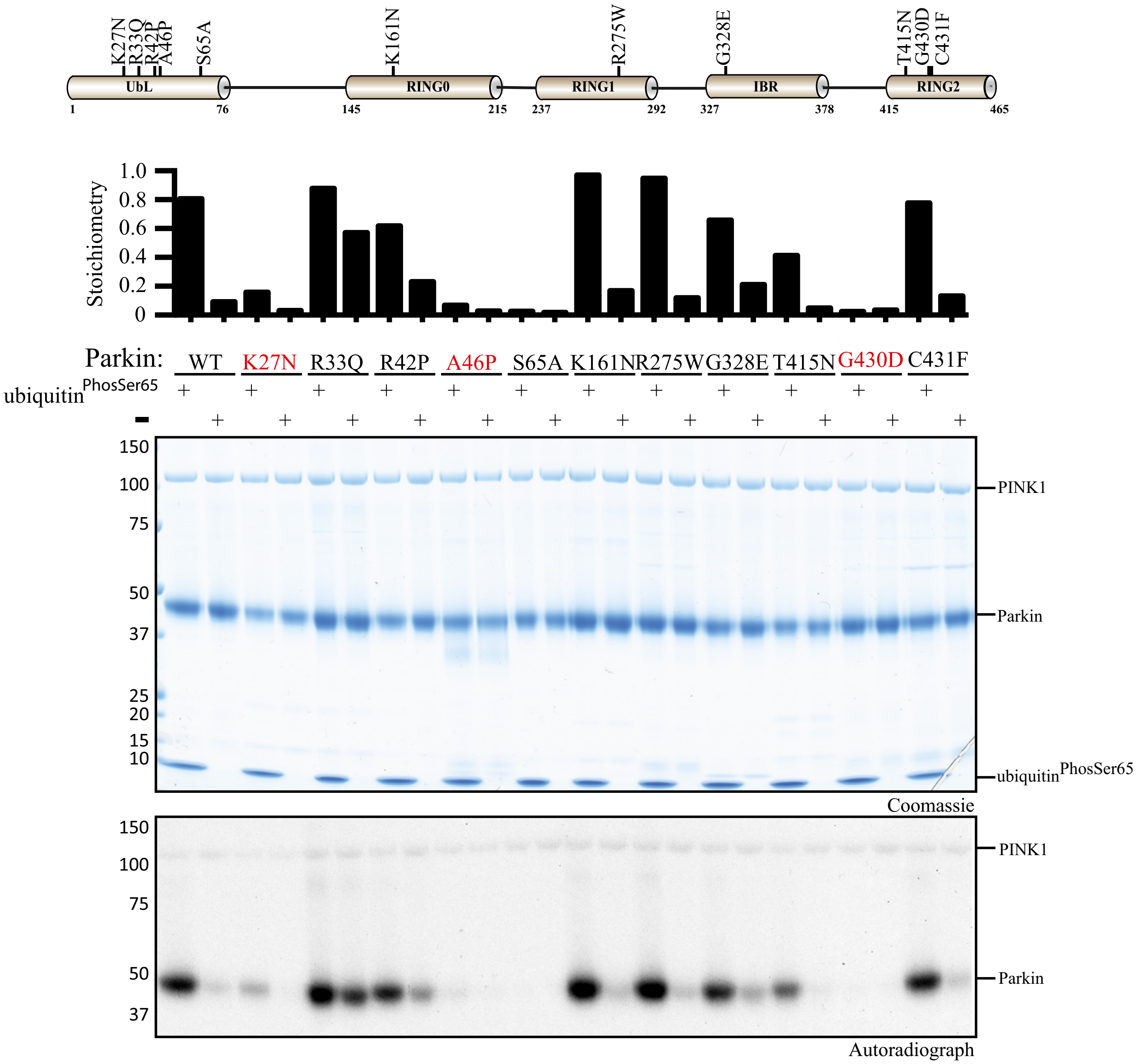

Figure EV4. Identification of Parkinson's disease-associated mutants that disrupt ubiquitin$^{Phospho-Ser65}$-enhanced phosphorylation of Parkin by TcPINK1.
Schematic of Parkin domain and location of disease-associated Parkin mutants (upper panel). Wild-type (WT) full-length Parkin or the indicated disease point mutant was incubated with wild-type MBP-TcPINK1 and Mg^{2+} [γ-^{32}P] ATP in the presence or absence of ubiquitin$^{Phospho-Ser65}$. Proteins were detected by Colloidal Coomassie Blue staining (top panel) and Parkin phosphorylation levels assessed by incorporation of [γ-^{32}P] ATP detected by autoradiography (bottom panel) and displayed above the panel (lower Panel).

 © 2015 The Authors

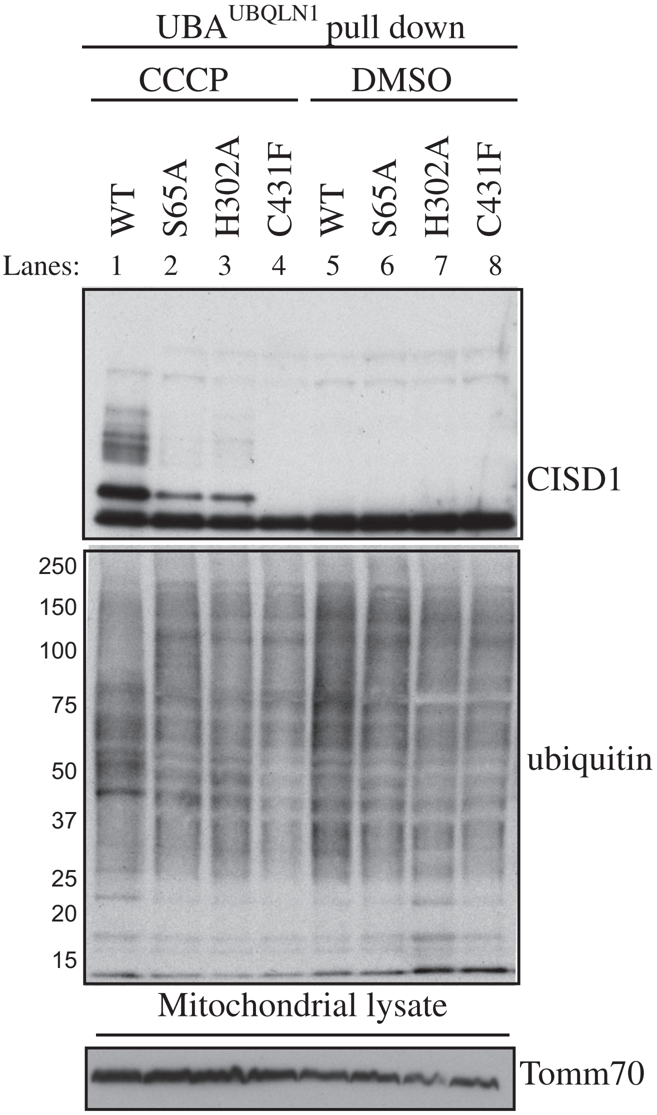

Figure EV5. Parkin His302 is required for optimal activation of Parkin ubiquitin E3 ligase activity at mitochondria in response to PINK1 activation by CCCP.

Wild-type HeLa cells were transfected with untagged wild-type (WT), and Ser65Ala (S65A)-, His302Ala (H302A)- or Cys431Phe (C431F)-mutant Parkin and stimulated with 10 μM of CCCP or DMSO for 6 h. Mitochondrial enriched extracts were incubated with a ubiquitin-binding resin derived from His-Halo-Ubiquilin UBA-domain tetramer (UBA^{UBQLN1}). Captured ubiquitylated proteins were subjected to immunoblotting with CISD1 and ubiquitin antibodies. In parallel, mitochondrial input extracts were immunoblotted with Tomm70 antibody.

© 2015 The Authors
