# Supplementary material for: Binding to serine 65-phosphorylated ubiquitin primes Parkin for optimal PINK1-dependent phosphorylation and activation
Source: EMBO Rep. 2015 Jun 26;16(8):939–54. doi: 10.15252/embr.201540352 (PMC4552487; doi:10.15252/embr.201540352)
Supplement: Supplementary file 3 [file embr0016-0939-sd3.pdf]

EMBO
reports

Manuscript EMBOR-2015-40352

Binding to Serine 65-phosphorylated ubiquitin primes Parkin for optimal PINK1-dependent phosphorylation and activation

Agne Kazlauskaite, Ruben J. Martinez-Torres, Scott Wilkie, Atul Kumar, Julien Peltier, Alba Gonzalez, Clare Johnson, Jinwei Zhang, Anthony G. Hope, Mark Peggie, Matthias Trost, Daan M.F. van Aalten, Dario R. Alessi, Alan R. Prescott, Axel Knebel, Helen Walden and Miratul M.K. Muqit

Corresponding author: Miratul M.K. Muqit, University Dundee

Review timeline:

Submission date:	11 March 2015
Editorial Decision:	30 March 2015
Revision received:	28 May 2015
Editorial Decision:	05 June 2015
Revision received:	09 June 2015
Accepted:	10 June 2015

Transaction Report:

(Note: With the exception of the correction of typographical or spelling errors that could be a source of ambiguity, letters and reports are not edited. The original formatting of letters and referee reports may not be reflected in this compilation.)

Editor: Nonia Pariente

1st Editorial Decision 30 March 2015

Many thanks for your patience while your study was peer-reviewed at EMBO reports. I apologize for the glitch in our system regarding the status of your manuscript. We indeed received the last referee report on Friday, and a referee cross-consultation was started, but no decision made or decision letter sent until now.

As I mentioned, we now have received three referee reports on your study, which you will find attached. Although all referees acknowledge the potential interest of your findings, they all also raise important issues regarding the conclusiveness of the findings. After additional consultation with the referees, it is clear that a successful revision would need to address the concerns raised regarding the effects that H302 (and K151) mutation might have on Parkin structure and function, address all major technical issues raised -including important control experiments to exclude other proposed roles of phosphorylated Ub-, and strengthen the functional data showing the importance of His302 for Parkin activity in cells (for Parkin translocation and ideally degradation of Parkin substrates).

Given that all referees provide constructive suggestions on how to make the work more conclusive, I would be happy to give you the opportunity to revise your manuscript. Please note that it is EMBO reports policy to undergo one round of revision only and thus, acceptance of your study will depend

on the outcome of the next, final round of peer-review.

I look forward to seeing a revised form of your manuscript when it is ready. In the meantime, please contact me if I can be of any assistance.

REFEREE REPORTS:

Referee #1:

Muqit and colleagues elucidate the mechanism of E3 ligase parkin activation by S65-phosphorylated ubiquitin. They show that Phospho-Ser65-modified ubiquitin directly binds His302 of parkin to prime its activation by PINK1-mediated phosphorylation. Mechanistically, P-Ser65 ubiquitin disrupts parkin autoinhibitory interaction between UBL and C-terminus to promote PINK1 access to Ser65 of parkin UBL. This finding adds another insight into activation of disease-related E3 ligase parkin. However, more evidence is needed to support specificity and relevance of parkin His302 interaction with P-Ser65 ubiquitin, in particular in light of other proposed models. Also, importance of this interaction in cells needs to be tested by analyzing degradation of various parkin substrates and mitophagy or in other functional assays.

Major comments:

1. Further characterization of the interaction surfaces is needed: the authors should identify more hydrophobic pocket residues involved in interactions or alternatively obtain some structural data to support these findings
2. Could His302 or K151 mutations unfold parkin and affect parkin activity independently of binding P-Ser65 Ub?
3. Do P-Ser65-modified Ub chains (for instance, di- or tetra-Ub) have the same effect as monomeric Ub in activating parkin?
4. Related to point 2; include His302 and K151 mutants into experiments in Figure 5. His302 mutation might activate parkin.
5. It is crucial to provide some functional data showing His302 importance for parkin activity in cells. For example, His302 mutant should be expressed in cells and parkin substrate degradation including proteins and mitochondria should be investigated to prove its relevance in vivo.

Minor comments:

1. Small molecular agents should not be mentioned in the abstract unless authors provide any data related to this application.
2. Page 5, 24th line; E2 ligase?
3. On Figure 2B, D, compare and indicate fold change between wt and affected or unaffected mutants to measure how 'partial' effects are.
4. Include K151 in your ITC measurements in Figure 3.
5. Are there any controls (on the same gel) for Figure 5A right panels?
6. Could authors discuss their model in relation to feed-forward mechanism of parkin activity in cells proposed by Harper and colleagues?
7. The drawing of the model on Figure 6 is suboptimal; make a simpler, more compact and self-explanatory drawing.

Referee #2:

Kazlauskaite and colleagues report that the activation of the ubiquitin ligase parkin by phospho-ubiquitin (pUb) occurs through the priming of parkin for phosphorylation by PINK1. PINK1 and Parkin have garnered a great deal of interest since the demonstration that they act together as a quality control system for mitochondria. Levels of PINK1, a protein kinase, increase upon mitochondrial depolarization. PINK1 then activates parkin to cause its translocation to mitochondria

© European Molecular Biology Organization

and activation. In 2012, Muqit's laboratory showed that parkin is phosphorylated on residue serine 65 of its Ubl domain and that this is a key step in the activation of parkin. Last year the laboratory was one of several that showed that PINK1 also phosphorylates ubiquitin and that phosphorylated ubiquitin can directly activate parkin.

The present paper adds important new elements to our understanding of this process. In a series of experiments looking at the phosphorylation of parkin, the authors show that parkin phosphorylation is strongly enhanced by the addition of pUb (Fig 1). The effect specific for parkin and is blocked by mutation of histidine residue H302 in parkin (Fig 2). This is confirmed by results in transfected cells where the mutation H302A strongly decreases parkin phosphorylation (Fig 3B). Using isothermal titration calorimetry and Alphascreen, the authors show that pUb binds to parkin and that it competes with Ubl binding binding to deltaUbl-parkin (Fig 3A and 4). Finally, they show that phosphorylated parkin is not further stimulated by the addition of pUb.

While missing a mechanistic explanation for the key observation that pUb promotes parkin phosphorylation, the paper is a significant contribution to our understanding of parkin activation. My main concern is that the authors' two-step model of activation ignores the clear role of pUb even in the absence of PINK1. Promoting phosphorylation may be "a" major role of pUb binding but I don't think the authors can say it is "the" major role. For the most part, the authors have been careful in their wording but they should add a paragraph to acknowledge other roles of pUb binding. Comparison of the activity of parkin+pUb (Fig 5A, left panel, right-most lane) with the activity of phospho-parkin alone (Fig 5A right panel, left lane) shows that pUb binding can directly activate parkin even in the absence of PINK1. The authors' previous publication (Kazlauskaite et al, Biochem J, 2014) showed that pUb can stimulate S65A parkin as well as deltaUbl parkin. None of those observations are consistent with a pUb playing only a priming role for parkin phosphorylation.

Major points:

1) The authors need to add a discussion of the other roles of pUb in the activation of parkin. How did the authors assign a temporal ordering to their two-step model? Ordureau et al, 2014 have shown that phospho-parkin binds pUb with higher affinity than does unphosphorylated parkin. From that, they concluded that parkin phosphorylation is the first step in the activation while pUb binding is the second step. The authors should discuss whether both steps are necessary and if there is any evidence for a sequential ordering of the two steps.

Reword the sentence, "In particular, we have found that the major role of ubiquitin-pSer65" on page 9 to say "a major role".

2) A second major conclusion of the paper is that His302 plays a key role in pUb binding but the authors need to show that H302A parkin is fully active as a ubiquitin ligase when phosphorylated. The authors convincingly show H302A parkin does not bind pUb and that its phosphorylation and ubiquitin ligase activity are not stimulated by pUb. However, it is not clear whether this represents a complete loss of structure or is a specific loss of pUb-binding activity. The mutant appears to elute slightly earlier on size-exclusion chromatography (Suppl Fig 3), which would be consistent with unfolding or a small amount of aggregation. Suppl Fig 4 shows other mutations (e.g. G430D) block parkin phosphorylation. The authors suggest that these mutations may trap the protein in a "conformation in which the Ubl domain is less accessible". What is the evidence that H302A parkin is not similarly conformationally trapped and globally inactive? Can H302A parkin be phosphorylated to the same extent (70%) as wild-type parkin? Is it as active as a ubiquitin ligase?

3) The authors need to correct the description of the binding site of the Ubl domain (first paragraph on page 6). The crystal structure of full-length parkin shows the Ubl domain binds to RING1 (Trempe et al, 2013). The data in reference 11 (Chaugule et al, 2011) was based on binding of peptide fragments to the Ubl domain and is certainly less reliable. Further, the correct reference for the crystal structure of the isolated Ubl domain is missing - Tomoo et al, Biochim Biophys Acta. 2008, 1784(7-8):1059-67. Finally, it is incorrect to say that Ser65 is "buried in the hydrophobic core of the domain" (page 6, line 4). The serine residue is in a loop.

Minor points:

4) The ubiquitination assay with Miro1 (Fig 2C) has unequal loadings and isn't equal to the high quality of the other results in the paper. While I appreciate that it is the ratio with and without pUb that is important, the figure would be more convincing if the experiment were rerun.

5) The font site in the insets in ITC experiments of Fig 3A is too small and the units (cal, molar etc) are missing. Given that the delta H is larger for the H302A parkin than wild-type parkin, why is the noise level so much higher? The integrated values show that both experiments have roughly 8 kcal of heat absorbed per mole of pUb added yet the actual data curves show a much weaker signal for the H302A mutant. Were the protein concentrations the same in both experiments?

6) The description of Suppl Fig 3 needs to be improved in the text (page 5, last paragraph). The reference to the Dac-tag system is confusing since it only relates to the preparation of phosphorylated Ub and not to the actual experiment. The text should indicate that size exclusion chromatography was used. In the suppl figure, the axes labels should be marked in the figure, not in the legend.

7) The authors claim that phosphorylated Ser65-Parkin is not further activated following addition of pUb. Is this lack of activation simply due to the fact that, under the conditions tested, parkin is already maximally active? The authors show the assay with diluted parkin is not saturated but that is not the same issue. Parkin fully saturated with pUb shows similar activity to fully phosphorylated parkin (Fig 5A). Doesn't this imply that either phosphorylation event fully activates parkin?

8) In figure 3B, the difference between lanes 1, 2 and 3 needs to be stated. Are they simply triplicates or did something vary? Why does the amount of phophorylated parkin increase?

Typos

Ref 29 is incomplete.

There are a number of inconsistent or incorrect capitalizations - Colloidal (pg 17), Flowrate (page 13), Mercaptoethanol (pg 11)

Referee #3:

EMBOR-2015-40352V1 Muqit
Parkin E3 ligase activity is activated by PINK1 by direct phosphorylation of Ser65 in its Ubiquitin-like (UBL) domain, presumably relieving autoinhibition. Interestingly, phosphorylation of ubiquitin itself at S65 by Pink1 is also proposed to be involved in Parkin ligase activation. Here, the authors provide evidence for a sequential phosphorylation priming model, in which phosphoSer65-ubiquitin binds to Parkin and promotes UBL S65 phosphorylation. They also showed that the E3 activity of purified S65 phosphorylated Parkin was not further activated by pSer65-Ub. Based on published Parkin structures, they identified a conserved residue, His302, located in a pocket in the C-terminal region of Parkin, which could potentially engage in pS65-Ub binding. Consistent with their hypothesis, mutation of His302 to Ala significantly reduced binding of pS65-Ub to Parkin, and also decreased phosphorylation of S65 in Parkin following mitochondrial uncoupler treatment of cells. Overall, this study contains careful and well presented work, with convincing experimental data that provides new mechanistic insights into Parkin E3 ligase activation by pS65-Ub, which suggest that a pocket in the C-terminal part of Parkin containing His302 provides a docking site for pS65-Ub leading to displacement of the N-terminal Parkin UBL domain from an autoinhibitory binding mode. However, the following points need to be addressed before the model can be firmly established.
1. Figure 1A: The authors should include Parkin S65D and UBLΔ Parkin as controls in this kinase assay. In their previous work using an in vitro kinase assay with Parkin and PINK1 alone, they showed that S65 is the major phosphorylation site in Parkin. However, in this new assay with pS65-Ub, Parkin phosphorylation was substantially increased over that observed in the absence of pS65-Ub. This might simply reflect increased stoichiometry of S65 phosphorylation, but it could also indicate that the observed Parkin phosphorylation occurred at sites other than S65, which may have gone undetected by MS. Phosphoubiquitin binding might provide a new docking site for PINK1 to

phosphorylate Parkin at additional sites. The authors did use S65A Parkin (Figure S4), but the inclusion of Parkin S65D (an open mutant form of Parkin) and Parkin lacking the UBL domain, could be used to confirm that PINK1 only phosphorylates Parkin at S65 in the presence of pS65-Ub.
2. Figure 2: The identification of H302 as a key surface residue for pS65-Ub binding is an important advance. However, the approaches used to conclude that H302 is a phosphate contact residue in bound pS65-Ub are largely indirect, and do not exclude the possibility that H302 is critical for maintaining a proper structure of the RBR domain, such that Parkin H302A adopts a structure in which S65 is less accessible. In line with this concern, G430D also affected Parkin phosphorylation stimulated by pS65-Ub (Figure S4).
The authors showed that Parkin H302A E3 ligase activity was not activated by pS65-Ub (Figure 2C). However, it is possible that the H302A mutation per se cripples Parkin E3 activity. Therefore, it is important to demonstrate that Parkin UBLΔ H302A has the same E3 activity as Parkin UBLΔ WT.
3. Figure 3B: It is known that the mitochondrial translocation of Parkin in response to mitochondrial damage depends on its E3 activity. Therefore, if Parkin H302A has intrinsically decreased E3 activity, Parkin mitochondrial translocation would be reduced, and as a result one would expect to see a decrease in S65 phosphorylation because Parkin would be unable to gain access to PINK1 on the mitochondrion. Whether or not the H302A mutation affects Parkin mitochondrial translocation should be discussed in the manuscript.
4. Figure 4: pS65-UBL was much less effective at inhibiting the GST-UBL/UBLΔ Parkin interaction than pS65-Ub, but a IC50 could be measured for pS65-UBL binding. This implies that pS65-UBL cannot bind to Parkin. Why this should pS65-UBL bind less well than pS65-Ub? If the actual or predicted structures of pS65-Ub and pS65-UBL are docked into the putative Pocket 2 site on Parkin that contains H302, can the authors observe a possible steric clash that might either prevent or lower the affinity of pS65-UBL binding. Despite the large difference in affinity of pS65 Ub and pS65-UBL for binding to Parkin, in the context of the full-length Parkin protein the local concentration of pS65-UBL will be effectively infinite, and therefore the UBL domain might still be able to bind to the pS65-Ub-binding site on Parkin once it is phosphorylated at S65 despite the lower affinity. If pS65-UBL does bind to the pS65-Ub site intramolecularly, then this could provide a mechanism through which phosphorylation of S65 in the UBL leads to constitutive activation of the Parkin E3 ligase, which is what the assay data in Figure 5 indicate. This would then mean that the model in Figure 6 is inaccurate, since intramolecular binding of pS65-UBL could displace pS65-Ub, leading to the active open Parkin configuration.
Minor points: 1. Page 7/Figure 4: GST is a constitutive dimer, and therefore GST-UBL will be dimeric, and this will result in increased avidity for any UBL binding partner. This needs to be taken into consideration
2. Page 7: Using "GST-UBL-Parkin" as a way to describe the interaction between GST-UBL and UBLΔ Parkin is confusing, because the dash in GST-UBL means that GST is linked to the UBL covalently, whereas the dash between GST-UBL and Parkin means a noncovalent association. It would be better if this were spelled out in the text.
3. Figure 4: It would be easier for the reader appreciate the data in this figure, if there were an accompanying table listing the measured IC50's for the various competition assays.

1st Revision - authors' response | 28 May 2015

Major Points raised by Reviewer 1

1. Further characterization of the interaction surfaces is needed: the authors should identify more hydrophobic pocket residues involved in interactions or alternatively obtain some structural data to support these findings

We agree with Reviewer 1 that it would be interesting to identify more residues on Parkin required for P-Ser65 Ub interaction. However, only structural analysis of P-Ser65 Ub bound to Parkin would provide decisive insights into these additional residues, which we believe is beyond the scope of this paper.

2. Could His302 or K151 mutations unfold parkin and affect parkin activity independently of binding P-Ser65 Ub?

We have undertaken thermal stability analysis and this is now included in Figure EV4A. The thermal stability data show that the H302A and K151A mutants of Parkin are as stable as wild-type Parkin, with no measurable change in thermal stability. This indicates no significant perturbation of fold or structural integrity.

Furthermore, since Parkin can be activated by direct phosphorylation of Parkin at Ubl Ser65 independent of P-Ser65 Ub, we have undertaken analysis of full-length Parkin H302A E3 ligase activity with a non-phosphorylatable S65A mutant of ubiquitin and demonstrate that this exhibits similar activity to wild-type Parkin. This data is now included in Figures EV4B and EV13.

Overall these additional analyses support our conclusions that the effects of H302A and K151A mutants we observe are due to loss of binding to P-Ser65 Ub and not simply due to protein unfolding.

3. Do P-Ser65-modified Ub chains (for instance, di- or tetra-Ub) have the same effect as monomeric Ub in activating parkin?

We have investigated whether P-Ser65 Ub modified ubiquitin chains would have the same effect as monomeric ubiquitin in promoting Parkin phosphorylation by PINK1. Addition of ubiquitin dimers of each linkage type (Met1, Lys6, Lys11, Lys27, Lys 29, Lys33, Lys48 and Lys63) or ubiquitin tetramers with Met1, Lys6, Lys11, Lys 29, Lys33, Lys48 and Lys63 linkages led to a similar enhancement of Parkin phosphorylation by PINK1 as that observed following addition of monomeric ubiquitin. This data is now included in Figure EV3A. In addition we have explored whether P-Ser65 Ub attached to a model substrate (Dac-Ubiquitin) would have the same effect and indeed observe similar activation of Parkin as monomeric ubiquitin. This data is now included in Figure EV3B.

4. Related to point 2; include His302 and K151 mutants into experiments in Figure 5. His302 mutation might activate parkin.

We have generated Parkin H302A mutant protein that is approximately ~90% phosphorylated at Ser65. We have undertaken E3 ligase assays alongside wild-type Parkin that is similarly phosphorylated at Ser65 and this demonstrates similar activity suggesting that the H302A mutation does not unfold Parkin. We have now included this data in Figure EV13.

5. It is crucial to provide some functional data showing His302 importance for parkin activity in cells. For example, His302 mutant should be expressed in cells and parkin substrate degradation including proteins and mitochondria should be investigated to prove its relevance in vivo.

Upon mitochondrial depolarisation in HeLa cells, we have assessed Parkin E3 ligase activity in cells by monitoring ubiquitylation of the Parkin substrate CISD1. In our cell based assay we observe robust ubiquitylation of CISD1 by wild-type Parkin

© European Molecular Biology Organization

and no activity upon expression of the catalytic-inactive disease mutant of Parkin, C431F. Consistent with our in vitro analysis, we observe significant loss of Parkin activity upon expression of the H302A mutant suggesting that His302 is critically required for maximal Parkin activation in cells upon PINK1 activation induced by mitochondrial depolarisation. We have included this data in Figure EV8.

Minor points raised by Reviewer 2:

1. Small molecular agents should not be mentioned in the abstract unless authors provide any data related to this application.

We agree with the reviewer and have now changed the sentence to: "This study provides new mechanistic insights into how Parkin is activated by ubiquitin$^{Phospho-Ser65}$ and will aid in the development of small molecule activators of Parkin that mimic the effect of ubiquitin$^{Phospho-Ser65}$."

2. Page 5, 24th line; E2 ligase?

We have now amended this sentence to "…..to stimulate the discharge of the ubiquitin-loaded E2, UbcH7, by Parkin."

3. On Figure 2B, D, compare and indicate fold change between wt and affected or unaffected mutants to measure how 'partial' effects are.

We have added the fold changes into Figure 2B above the bar chart. Given the impact of point mutants to Parkin stimulated E2 discharge in Figure 2D, we do not believe that providing fold changes to Figure 2D would add significantly to the figure.

4. Include K151 in your ITC measurements in Figure 3.

We have repeated ITC analysis for wild-type Parkin in parallel with the H302A and K151A mutants. This reveals that the K151A mutant binds P-Ser65 Ub with 45-fold lower affinity (Kd of ~7μM) than wild-type Parkin whilst the H302A mutant binds P-Ser65 Ub with 265-fold lower affinity. We have revised Figure 3 to include this new data.

5. Are there any controls (on the same gel) for Figure 5A right panels?

The immunoblots were all undertaken in parallel. Gels were run and transferred to membrane in parallel. All membranes were probed with primary and secondary antibodies in parallel and developed in parallel with ECL. We have adjusted the figure to reflect this by adding dotted lines. Note that Figure 5A is now 6A in the revised version.

In addition we include at the end of this letter the source image of the original ECL exposed film that was scanned in to generate Figure 6A. This has been included at the end of this letter as Appendix 1 for the reviewer's information.

Furthermore, the data in Figure EV13 (addressing the role of H302A

phosphorylated Parkin) also provides all the required controls in the same figure.

6. Could authors discuss their model in relation to feed-forward mechanism of parkin activity in cells proposed by Harper and colleagues?

We have now expanded the conclusion to address this. The text added to the conclusion is as follows:

"Recent models of Parkin activation by PINK1 have highlighted a role for ubiquitin$^{\text{Phospho-Ser65}}$ in binding and recruiting phosphorylated Parkin to mitochondria triggering a feed-forward amplification loop of Parkin activity at the mitochondria [19, 21, 29]. The data presented here demonstrates that another major role of ubiquitin$^{\text{Phospho-Ser65}}$ is to prime Parkin Ubl Ser^{65} for phosphorylation by PINK1. Furthermore, we have found that once phosphorylated, Parkin$^{\text{Phospho-Ser65}}$, is no longer sensitive to ubiquitin$^{\text{Phospho-Ser65}}$ suggesting that the E3 ligase activity of Parkin may largely be driven by PINK1 mediated phosphorylation of the Ubl domain of Parkin at Ser^{65} that may stabilise it in the active conformation. Our findings elaborate a 2-step model of Parkin activation by ubiquitin$^{\text{Phospho-Ser65}}$ and the PINK1 kinase (Figure 7) that offers novel insights into how Parkin is activated and adds another layer of interdependence between Parkin Ubl $^{\text{Phospho-Ser65}}$ and ubiquitin$^{\text{Phospho-Ser65}}$ that would be predicted to drive feed-forward amplification of Parkin E3 ligase activity."

7. The drawing of the model on Figure 6 is suboptimal; make a simpler, more compact and self-explanatory drawing.

We have made further changes to the model that we believe depict the 2-step activation process in a clear way. This amended model is designated Figure 7.

Major Points raised by Reviewer 2:

1) The authors need to add a discussion of the other roles of pUb in the activation of parkin. How did the authors assign a temporal ordering to their two-step model? Ordureau et al, 2014 have shown that phospho-parkin binds pUb with higher affinity than does unphosphorylated parkin. From that, they concluded that parkin phosphorylation is the first step in the activation while pUb binding is the second step. The authors should discuss whether both steps are necessary and if there is any evidence for a sequential ordering of the two steps.

Reword the sentence, "In particular, we have found that the major role of ubiquitin-pSer65" on page 9 to say "a major role".

We fully accept this excellent point that ubiquitin-pSer65 is likely to have additional roles and have changed the text accordingly to state that we have found "a major role" for ubiquitin-pSer65. Furthermore, we expanded the conclusion to address this point as follows:

"Recent models of Parkin activation by PINK1 have highlighted a role for

© European Molecular Biology Organization

ubiquitin$^{Phospho\text{-}Ser65}$ in binding and recruiting phosphorylated Parkin to mitochondria triggering a feed-forward amplification loop of Parkin activity at the mitochondria [19, 21, 29]. The data presented here demonstrates that another major role of ubiquitin$^{Phospho\text{-}Ser65}$ is to prime Parkin Ubl Ser^{65} for phosphorylation by PINK1. Furthermore, we have found that once phosphorylated, Parkin$^{Phospho\text{-}Ser65}$, is no longer sensitive to ubiquitin$^{Phospho\text{-}Ser65}$ suggesting that the E3 ligase activity of Parkin may largely be driven by PINK1 mediated phosphorylation of the Ubl domain of Parkin at Ser^{65} that may stabilise it in the active conformation. Our findings elaborate a 2-step model of Parkin activation by ubiquitin$^{Phospho\text{-}Ser65}$ and the PINK1 kinase (Figure 7) that offers novel insights into how Parkin is activated and adds another layer of interdependence between Parkin Ubl $^{Phospho\text{-}Ser65}$ and ubiquitin$^{Phospho\text{-}Ser65}$ that would be predicted to drive feed-forward amplification of Parkin E3 ligase activity."

2) A second major conclusion of the paper is that His302 plays a key role in pUb binding but the authors need to show that H302A parkin is fully active as a ubiquitin ligase when phosphorylated. The authors convincingly show H302A parkin does not bind pUb and that its phosphorylation and ubiquitin ligase activity are not stimulated by pUb. However, it is not clear whether this represents a complete loss of structure or is a specific loss of pUb-binding activity.
The mutant appears to elute slightly earlier on size-exclusion chromatography (Suppl Fig 3), which would be consistent with unfolding or a small amount of aggregation. Suppl Fig 4 shows other mutations (e.g. G430D) block parkin phosphorylation. The authors suggest that these mutations may trap the protein in a "conformation in which the Ubl domain is less accessible". What is the evidence that H302A parkin is not similarly conformationally trapped and globally inactive? Can H302A parkin be phosphorylated to the same extent (70%) as wild-type parkin? Is it as active as a ubiquitin ligase?

This point is broadly similar to what has been raised by Reviewer 1 (Point 2). Please refer to our response to that point in which we have undertaken further analysis to exclude the possibility that the Parkin H302A mutant is unfolded. This includes the demonstration that H302A Parkin can be phosphorylated to the same extent as wild-type Parkin and exhibit similar activity and this data has been included in Figure EV13.

We do not agree with the reviewer's interpretation that the Parkin H302A mutant elutes earlier on size exclusion chromatography – the difference is not significant and the peaks overlay within an appropriate margin of error. Taken together with our extensive analysis on stability and activity of H302A Parkin, we conclude that this mutant is likely to be folded similar to wild type Parkin.

3) The authors need to correct the description of the binding site of the Ubl domain (first paragraph on page 6). The crystal structure of full-length parkin shows the Ubl domain binds to RING1 (Trempe et al, 2013). The data in reference 11 (Chaugule et al, 2011) was based on binding of peptide fragments to the Ubl domain and is certainly less reliable. Further, the correct reference for the crystal structure of the isolated Ubl domain is missing - Tomoo et al, Biochim Biophys Acta. 2008, 1784(7-8):1059-67. Finally, it is incorrect to say that Ser65 is

"buried in the hydrophobic core of the domain" (page 6, line 4). The serine residue is in a loop.

We have revised the text to incorporate these suggestions and included the reference Tomoo et al (Ref 32). The revised text is as follows:

"We next investigated the mechanism by which ubiquitin$^{\text{Phospho-Ser65}}$ enhances PINK1-dependent phosphorylation of Parkin Ubl Ser^{65}. The Ubl domain binds to the RING1 domain [12] and can also interact with Parkin lacking the Ubl domain *in trans* [11]. Crystal and NMR solution structures of the Ubl domain reveal that Ser^{65} resides in a loop adjacent to the fifth β-strand that exhibits conformational flexibility [30-32] and may only be partially surface accessible in the full length structure. We therefore hypothesised that ubiquitin$^{\text{Phospho-Ser65}}$ binding might disrupt the ability of the Ubl domain to bind to the C-terminal domain and therefore render Ser^{65} of the Ubl domain to be more accessible for phosphorylation by PINK1."

Minor Points raised by Reviewer 2

4) The ubiquitination assay with Miro1 (Fig 2C) has unequal loadings and isn't equal to the high quality of the other results in the paper. While I appreciate that it is the ratio with and without pUb that is important, the figure would be more convincing if the experiment were rerun.

We have now rerun the experiment and revised Figure 2C accordingly.

5) The font site in the insets in ITC experiments of Fig 3A is too small and the units (cal, molar etc) are missing. Given that the delta H is larger for the H302A parkin than wild-type parkin, why is the noise level so much higher? The integrated values show that both experiments have roughly 8 kcal of heat absorbed per mole of pUb added yet the actual data curves show a much weaker signal for the H302A mutant. Were the protein concentrations the same in both experiments?

We have repeated ITC analysis for wild-type Parkin in parallel with the H302A and K151A mutants. This reveals that the K151A mutant binds P-Ser65 Ub with 45-fold lower affinity (Kd of ~7μM) than wild-type Parkin whilst the H302A mutant binds P-Ser65 Ub with 265-fold lower affinity. We have removed the small inset and replaced this with a Table in Figure 3B that is of appropriate font size and contains all the appropriate units. In our new experiments the delta H of the wild-type and mutant Parkin proteins are similar. Furthermore, equivalent protein concentrations were used in all experiments. The thermodynamics of the reactions between P-Ser65 Ub and wild-type parkin or mutant Parkin indicate, in general, an entropically driven reaction (-TΔS); favorable entropy is an indication of changes in hydrophobic interaction and conformational changes. We have revised Figure 3 to include this new data and summarise –TΔS values in a Table.

© European Molecular Biology Organization

6) The description of Suppl Fig 3 needs to be improved in the text (page 5, last paragraph). The reference to the Dac-tag system is confusing since it only relates to the preparation of phosphorylated Ub and not to the actual experiment. The text should indicate that size exclusion chromatography was used. In the suppl figure, the axes labels should be marked in the figure, not in the legend.

In the revised manuscript Suppl Fig 3 is now Figure EV6 and we have revised the figure to mark axes labels in the figure. We have also added analysis of the Parkin Ser65Ala mutant to demonstrate that this is able to bind pUb in a complex. In relation to the data of Fig EV6, we have revised the text as follows:

"We also undertook complementary gel filtration analysis to assess for heterodimeric complex formation of wild-type Parkin with ubiquitin$^{Phospho-Ser65}$. We exploited the above-described N-terminal Dac-tag ubiquitin since this retains monomeric properties and enables visualisation of binding due to a large change in molecular weight that is easily visible by gel filtration analysis [27]. We next produced a tagged-fusion protein of Dac-ubiquitin$^{Phospho-Ser65}$ or non-phosphorylated ubiquitin. Size exclusion chromatography using a Superdex 200 Increase (10/300 GL) column was employed with 250 µl of purified protein at a concentration of 0.8 mg/ml. Incubation of Dac-ubiquitin with wild-type Parkin revealed no strong interaction and two separate peaks (Figure EV6E). In contrast we observed binding of wild-type Parkin with Dac-ubiquitin^{PhosSer65} with a shift in the size of the peaks indicating heterodimer formation (Figure EV6F). We also observed binding of a Parkin S65A mutant with Dac-ubiquitin^{PhosSer65} (Figure EV6K). Consistent with our ITC data, we observed no evidence of binding of the Parkin H302A mutant and Dac-ubiquitin^{PhosSer65} as judged by 2 separate peaks (Figure EV6G)."

7) The authors claim that phosphorylated Ser65-Parkin is not further activated following addition of pUb. Is this lack of activation simply due to the fact that, under the conditions tested, parkin is already maximally active? The authors show the assay with diluted parkin is not saturated but that is not the same issue. Parkin fully saturated with pUb shows similar activity to fully phosphorylated parkin (Fig 5A). Doesn't this imply that either phosphorylation event fully activates parkin?

We have now undertaken a timecourse analysis of phosphorylated Ser65-Parkin ubiquitin E3 ligase activity and demonstrate that at 5 min in which activity is not saturated, there is no further enhancement of phospho-Parkin activity upon addition of pUb. This new data is included as Figure EV12. Furthermore, whilst our immunoblots of ubiquitylation using ECL are not quantitative, we do not agree with the interpretation that the activity of Parkin fully saturated pUb is similar to phosphorylated Parkin. Qualitatively, using Miro1 ubiquitylation as a readout of Parkin activity, it is evident that phosphorylated Parkin is more active as seen in Figure 6A.

8) In figure 3B, the difference between lanes 1, 2 and 3 needs to be stated. Are they simply triplicates or did something vary? Why does the amount of phosphorylated parkin increase?

Figure 3B is now Figure 4A in the revised paper. Lanes 1-3 of Figure 4A represents

triplicates. This has been stated in the Figure legend.

Ref 29 is incomplete.

A complete reference is now provided and it is now 37.

There are a number of inconsistent or incorrect capitalizations - Colloidal (pg 17), Flowrate (page 13), Mercaptoethanol (pg 11)

These incorrect capitalisations have now been corrected for flowrate and mercaptoethanol. However, we have kept Colloidal as capitals.

Major Points raised by Reviewer 3:

1. Figure 1A: The authors should include Parkin S65D and UBLΔ Parkin as controls in this kinase assay. In their previous work using an in vitro kinase assay with Parkin and PINK1 alone, they showed that S65 is the major phosphorylation site in Parkin. However, in this new assay with pS65-Ub, Parkin phosphorylation was substantially increased over that observed in the absence of pS65-Ub. This might simply reflect increased stoichiometry of S65 phosphorylation, but it could also indicate that the observed Parkin phosphorylation occurred at sites other than S65, which may have gone undetected by MS. Phosphoubiquitin binding might provide a new docking site for PINK1 to phosphorylate Parkin at additional sites. The authors did use S65A Parkin (Figure S4), but the inclusion of Parkin S65D (an open mutant form of Parkin) and Parkin lacking the UBL domain, could be used to confirm that PINK1 only phosphorylates Parkin at S65 in the presence of pS65-Ub.

We thank the reviewer for this interesting point. We have repeated phosphorylation assays with S65A and UBLΔ Parkin and do not observe any phosphorylation of these Parkin species in the presence of p-S65-Ub indicating that under the assay conditions used S65 is the major phosphorylation site of Parkin. This new data has been included in Figure EV1.

2. Figure 2: The identification of H302 as a key surface residue for pS65-Ub binding is an important advance. However, the approaches used to conclude that H302 is a phosphate contact residue in bound pS65-Ub are largely indirect, and do not exclude the possibility that H302 is critical for maintaining a proper structure of the RBR domain, such that Parkin H302A adopts a structure in which S65 is less accessible. In line with this concern, G430D also affected Parkin phosphorylation stimulated by pS65-Ub (Figure S4).

This point is broadly similar to what has been raised by Reviewer 1 (Point 2). Please refer to our response to that point in which we have undertaken further analysis to exclude the possibility that the Parkin H302A mutant is unfolded. This includes the demonstration that H302A Parkin can be phosphorylated to the same extent as wild-type Parkin and exhibit similar activity and this data has been included in Figure EV13.

The authors showed that Parkin H302A E3 ligase activity was not activated by pS65-Ub (Figure 2C). However, it is possible that the H302A mutation per se

© European Molecular Biology Organization

cripples Parkin E3 activity. Therefore, it is important to demonstrate that Parkin UBLΔ H302A has the same E3 activity as Parkin UBLΔ WT.

We have undertaken E3 ligase assays with the Parkin UBLΔ H302A mutant and demonstrate that this exhibits similar basal constitutive activity as wild-type Parkin. This new data is included in Figure EV4B.

3. Figure 3B: It is known that the mitochondrial translocation of Parkin in response to mitochondrial damage depends on its E3 activity. Therefore, if Parkin H302A has intrinsically decreased E3 activity, Parkin mitochondrial translocation would be reduced, and as a result one would expect to see a decrease in S65 phosphorylation because Parkin would be unable to gain access to PINK1 on the mitochondrion. Whether or not the H302A mutation affects Parkin mitochondrial translocation should be discussed in the manuscript.

We have undertaken immunofluorescence analysis of Phospho-Ser65 Parkin in HeLa cells upon mitochondrial depolarisation. Upon expression of wild-type Parkin, we observe robust accumulation of Phospho-Ser65 Parkin at mitochondria upon mitochondrial depolarisation. Strikingly upon expression of H302A Parkin, we observe dramatic loss of Phospho-Ser65 signal at the mitochondria and instead observe cytosolic signal consistent with recent work from other groups that P-Ser65 Ub is required for Parkin recruitment to mitochondria and our conclusion that H302A disrupts this interaction. We have included this data in Figure 4B.

4. Figure 4: pS65-UBL was much less effective at inhibiting the GST-UBL/UBLΔ Parkin interaction than pS65-Ub, but a IC50 could be measured for pS65-UBL binding. This implies that pS65-UBL cannot bind to Parkin. Why this should pS65-UBL bind less well than pS65-Ub? If the actual or predicted structures of pS65-Ub and pS65-UBL are docked into the putative Pocket 2 site on Parkin that contains H302, can the authors observe a possible steric clash that might either prevent or lower the affinity of pS65-UBL binding. Despite the large difference in affinity of pS65 Ub and pS65-UBL for binding to Parkin, in the context of the full-length Parkin protein the local concentration of pS65-UBL will be effectively infinite, and therefore the UBL domain might still be able to bind to the pS65-Ub-binding site on Parkin once it is phosphorylated at S65 despite the lower affinity. If pS65-UBL does bind to the pS65-Ub site intramolecularly, then this could provide a mechanism through which
phosphorylation of S65 in the UBL leads to constitutive activation of the Parkin E3 ligase, which is what the assay data in Figure 5 indicate. This would then mean that the model in Figure 6 is inaccurate, since intramolecular binding of pS65-UBL could displace pS65-Ub, leading to the active open Parkin configuration.

Incomplete inhibition curves are generated by adding pS65-UBL to both wt and H302A parkin in Alphascreen assays. It is noted that these incomplete curves are shifted around 10 fold from the full curves generated by the addition of non-phospho-UBL. As mentioned in the text the pS65-UBL sample was 90% phosphorylated and 10% non-phosphorylated. This 10% non phosphorylated component could fully account for the inhibition in the pS65-UBL Alphascreen. Therefore, although we cannot entirely discount the possibility of an intramolecular interaction after UBL phosphorylation the Alphascreen data does

not support the interaction of pS65-UBL and the H302 containing Pocket 2 site UBLΔ Parkin. However, our assay does not rule out binding of pS65-UBL to UBLΔ Parkin at a distinct site.

Our findings and model are consistent with the possibility that the pS65-Ub and pS65-UBL are not docked into the putative Pocket 2 site on Parkin and may actually bind mutually exclusive sites within Parkin. We believe that further structural analysis of pS65-UBL bound to Parkin in trans or alternatively the structure of the Phospho-Ser65 Parkin will provide decisive insights on the mode of binding of pS65-UBL on Parkin.

Minor points:

1. Page 7/Figure 4: GST is a constitutive dimer, and therefore GST-UBL will be dimeric, and this will result in increased avidity for any UBL binding partner. This needs to be taken into consideration

Since the beads in the Alphascreen are coated with protein there will be an avidity component (variable number of protein/protein interactions holding beads together to generate signal) to the signal generated independently of whether or not GST acts as a dimer. Importantly the signal generated is via interaction of GST UBL and UBLΔ Parkin, which is consistent throughout all the experiments with the different phosphorylated molecules.

2. Page 7: Using "GST-UBL-Parkin" as a way to describe the interaction between GST-UBL and UBLΔ Parkin is confusing, because the dash in GST-UBL means that GST is linked to the UBL covalently, whereas the dash between GST-UBL and Parkin means a noncovalent association. It would be better if this were spelled out in the text.

We have amended the text accordingly to “GST-UBL to Parkin”.

3. Figure 4: It would be easier for the reader appreciate the data in this figure, if there were an accompanying table listing the measured IC50's for the various competition assays.

We have now provided a Table as Figure 5E.

© European Molecular Biology Organization

Appendix I

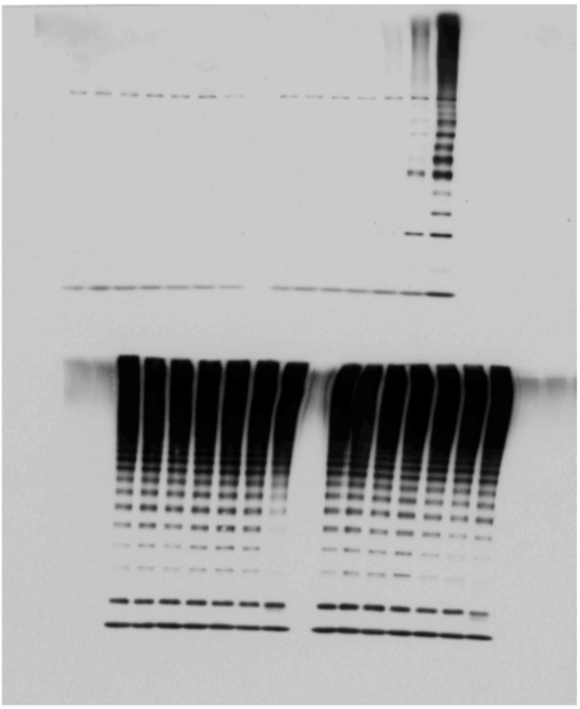

2nd Editorial Decision 05 June 2015

Thank you for your patience while we have reviewed your revised manuscript. I have now received feedback from referees 1 and 3, who were asked to assess your work. As you will see below, they are both now supportive of your study.

I am therefore very happy to write with an 'accept in principle' decision, which means that I will be happy to accept your manuscript for publication once a few minor issues/corrections have been addressed, as follows.

- There does not seem to be any statistical information in the relevant figure legends. Please go through your manuscript once more and ensure that all figures have been generated according to proper statistical analysis procedures, and all relevant figure legends include information on the number of independent experiments measured, the type of error bars used and statistical test applied to the data with values considered significant (if applicable). Information is missing in the legends of figures 1A, 1B, 2B, 2D and 5A-D (although data on the number of experiments performed is welcome for all figures).

- EMBO reports now accommodates the inclusion of extra figures (up to five) in the online version of the manuscript. These are presented in an expandable format inline in the main text, so that readers who are interested can access them directly as they read the article. They are also provided for download in a separate typeset PDF to accompany the Article PDF. These figures should be those of particular value to specialist readers, but which are not required to follow the main thread of the paper (and not additional controls or reagent optimization). They should be labeled expanded view. All other figures should be labeled as Appendix figure X, and their legends provided as a

separate word file; they will be included in a separate Appendix PDF.

After all remaining corrections have been attended to, you will receive an official decision letter from the journal accepting your manuscript for publication in the next available issue of EMBO reports. This letter will also include details of the further steps you need to take for the prompt inclusion of your manuscript in our next available issue.

We do not publish early version of articles before the proofs online, but our fast-track production process is 10 days from export to publication online.

Thank you for your contribution to EMBO reports.

REFEREE REPORTS:

Referee #1:

The authors have addressed all raised issues successfully. No further comments

Referee #3:

The authors have done a good job of addressing our concerns and those of the other reviewers. Ultimately, to understand exactly how pS65 Ub (but not pS65 UBL) interacts with H302/K151 in Parkin, additional structures will be needed. However, these are clearly beyond the scope of this paper, whose main goal was to define a possible binding site for pS65 Ub involved in activation of Parkin by PINK1.
The main new finding is that pS65 Ub binding to Parkin through an interaction requiring H302 primes the phosphorylation of the Parkin N-terminal UBL domain at S65 by PINK1, which displaces the UBL domain from its inhibitory interaction and results in Parkin in an open conformation that is active as an E3 ligase. This is suggests an additional role for pS65Ub in the activation of Parkin at damaged mitochondria that complements its role in recruiting Parkin to the mitochondrion. The data supporting their conclusion for this new role of pS65 Ub are convincing, and this finding should be published.
In summary, although one could suggest more experiments, I would now recommend publication of the paper without further revisions.

2nd Revision - authors' response 09 June 2015

Many thanks for that rapid decision made to our revised manuscript and inviting us to submit minor changes to our manuscript for final consideration for publication in EMBO Reports.
We have provided the required information on statistical analysis in all relevant Figure legends.
We have indicated those figures as EV and those that will be contained within the appendix.
We have made a slight amendment to the title as follows:

"Binding to Serine 65-phosphorylated ubiquitin primes Parkin for optimal PINK1-dependent phosphorylation and activation"

We have made a slight amendment to the abstract and changed "phospho-peptide binding" to "phosphoSer65-binding". We have uploaded the revised abstract online.

Many thanks for steering our manuscript so efficiently and ensuring its rigorous assessment that has enabled us to contribute what we believe will be a paper of significant interest to the field.

3rd Editorial Decision 10 June 2015

I am very pleased to accept your manuscript for publication in the next available issue of EMBO reports. We will also fast-track its advanced online publication. Thank you for your contribution to EMBO reports.

© European Molecular Biology Organization
